# Structural and chemical trapping of flavin‐oxide intermediates reveals substrate‐directed reaction multiplicity

**DOI:** 10.1002/pro.3879

**Published:** 2020-05-26

**Authors:** Kuan‐Hung Lin, Syue‐Yi Lyu, Hsien‐Wei Yeh, Yi‐Shan Li, Ning‐Shian Hsu, Chun‐Man Huang, Yung‐Lin Wang, Hao‐Wei Shih, Zhe‐Chong Wang, Chang‐Jer Wu, Tsung‐Lin Li

**Affiliations:** ^1^ Genomics Research Center Academia Sinica Taipei Taiwan; ^2^ The Institute of Biochemistry and Molecular Biology National Yang‐Ming University Taipei Taiwan; ^3^ Department of Food Science National Taiwan Ocean University Keelung Taiwan; ^4^ Biotechnology Center National Chung Hsing University Taichung City Taiwan

**Keywords:** Baeyer–Villiger oxidation, flavin mononucleotide, mandelate oxidase, monooxygenase, oxidative decarboxylation

## Abstract

Though reactive flavin‐N5/C4α‐oxide intermediates can be spectroscopically profiled for some flavin‐assisted enzymatic reactions, their exact chemical configurations are hardly visualized. Structural systems biology and stable isotopic labelling techniques were exploited to correct this stereotypical view. Three transition‐like complexes, the α‐ketoacid…N5‐FMN_ox_ complex (**I**), the FMN_ox_‐N5‐aloxyl‐C′α^−^‐C4α^+^ zwitterion (**II**), and the FMN‐N5‐ethenol‐N5‐C4α‐epoxide (**III**), were determined from mandelate oxidase (Hmo) or its mutant Y128F (monooxygenase) crystals soaked with monofluoropyruvate (a product mimic), establishing that N5 of FMN_ox_ an alternative reaction center can polarize to an ylide‐like mesomer in the active site. In contrast, four distinct flavin‐C4α‐oxide adducts (**IV**–**VII**) from Y128F crystals soaked with selected substrates materialize C4α of FMN an intrinsic reaction center, witnessing oxidation, Baeyer–Villiger/peroxide‐assisted decarboxylation, and epoxidation reactions. In conjunction with stopped‐flow kinetics, the multifaceted flavin‐dependent reaction continuum is physically dissected at molecular level for the first time.

## Abstract 2

PDB Code(s): 5ZZT, 6A24, 6A1W, 6A01, 6A1N, 6A1B, 6A0B, 6A36, 6A4H, 6A4G, 6A3D, and 7BSR

**ABBREVIATIONS**
BSA: bovine serum albumin
FAD: flavin adenine dinucleotide
FMN: flavin mononucleotide
FPLC: fast performance liquid chromatography
Hmo: hydroxy mandelate oxidase
LB: lysogeny broth
LC–MS: liquid chromatography‐mass spectrometry
NTA: nitrilotriacetic acid
PDA: photo diode array
WT: wild‐type

## 1 INTRODUCTION

**SCHEME 1:**
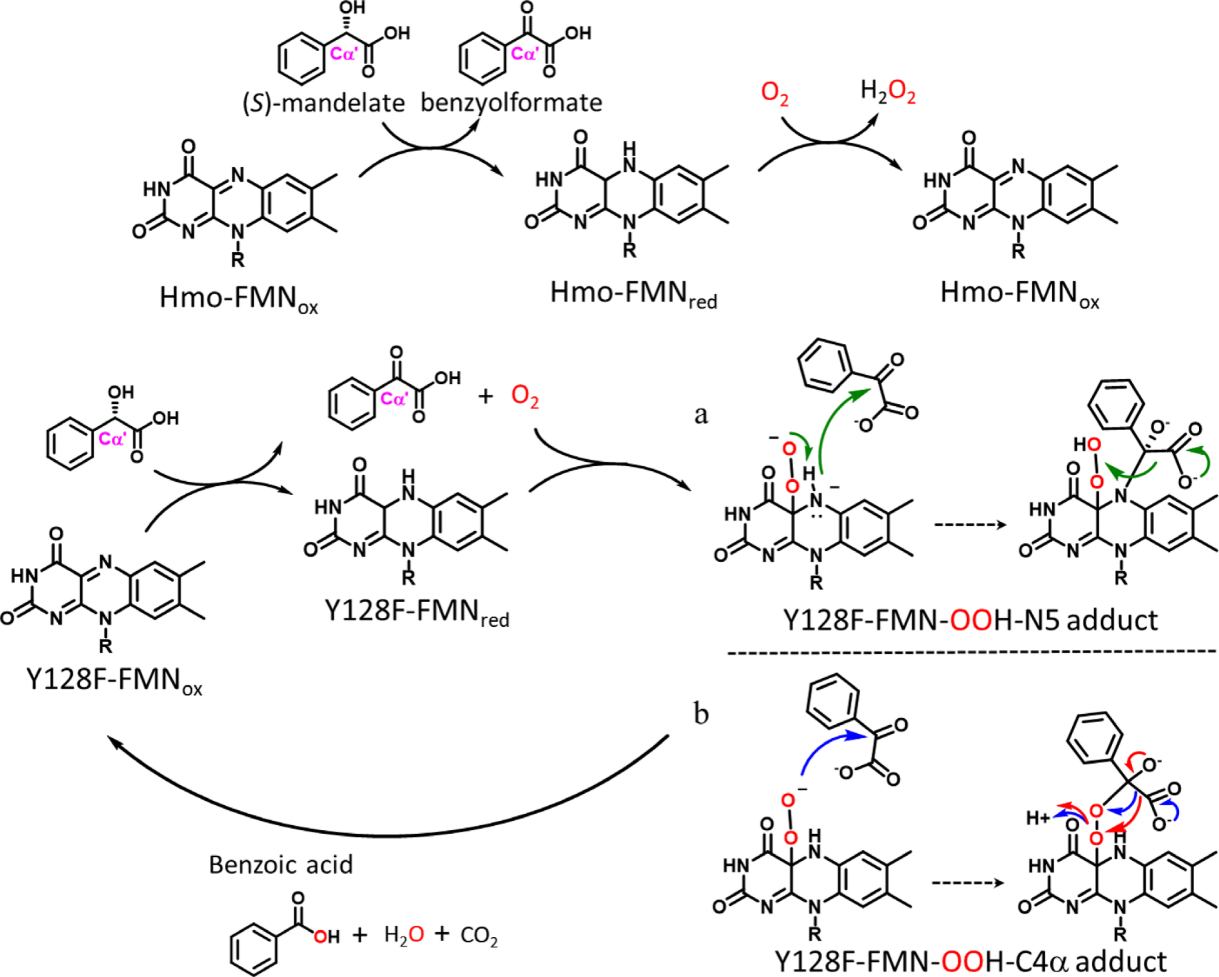
The reactions catalyzed by Hmo and its mutant Y128F. (a) Hmo oxidizes (*S*)‐mandelate to benzoylformate. (b) Y128F catalyzes the conversion of (*S*)‐mandelate en route benzoylformate to benzoate through a 4‐electron oxidative decarboxylation reaction, which recruits two possible intermediates a covalently linked alkyl‐N5 hydroperoxyl‐flavin adduct (A) or a C4α‐hydroperoxyl‐flavin adduct (B). Three different mechanisms are illustrated with green, blue or red arrows

The *p*‐hydroxy‐mandelate oxidase (Hmo) is a flavin mononucleotide (FMN)‐dependent oxidase catalyzing 2‐electron oxidation of α‐hydroxyacid to α‐ketoacid.^1^ The single mutant Y128F however acts like a monooxygenase catalyzing α‐hydroxyacid to decarboxylated acid, an unexpected 4‐electron oxidation reaction (Scheme 1).^1^ Both structural and biochemical interrogations have revealed that residue H252 is the committed base deprotonating α‐OH facilitating α‐hydride transfer, while residue Y128 acts as a ratchet leveraging the oxidation cascade by stabilizing C4α‐hydroperoxyflavin. Other than reduced FMN (FMN_red_) oxidized FMN (FMN_ox_) that possesses nucleophilicity was demonstrated recently in crystals of Y128F soaked with α‐ketoacid forming covalently‐linked‐flavin adducts.^2^ This fact underlined that under an active‐site perturbation an ylide‐like isoform (C4α^+^‐N5^−^) of FMN_ox_ is resulted, which is poised to nucleophilically attack α‐ketoacid to form N5‐acyl‐FMN_red_ upon decarboxylation. This phenomenon is attributed to reconfigured interactions amid flavin, active site residues, and substrates. The flavin isoalloxazine ring is arguably the most influencing factor, because it is ideally suited for oxidative or reductive reactions involving electron transfer to and from other redox‐active centers, as well as in reaction with the spin‐forbidden triplet‐state molecular oxygen (O_2_) via the charge‐transfer cadged system.^3^, ^4^, ^5^, ^6^ Flavoenzymes can be classified on the basis of the chemistry of the C4α‐hydroperoxy‐flavin intermediate. For NAD(P)H‐dependent monooxygenase, the C4α‐O‐O bond is broken with one oxygen atom incorporation to the substrate and the other release as water. For oxidase, C4α‐OOH is eliminated as free hydrogen peroxide (H_2_O_2_).^7^ Despite C4α‐hydroperoxy‐flavin an active intermediate, spectroscopic/kinetic methods have been established for some flavin‐containing monooxygenases.^8^, ^9^, ^10^, ^11^, ^12^, ^13^, ^14^ In general, C4α‐oxide is so unstable to be observed in oxidases, where the formation of the short‐lived C4α‐hydroperoxy intermediate may be mediated through an outer‐sphere electron transfer mechanism.^15^ Latest progresses are summarized as follows: A C4α‐hydroperoxy‐flavin species was identified in a fungal pyranose dehydrogenase.^16^ A flavin C4α‐adduct was spectroscopically detected in a crystalline state for a choline oxidase.^9^ An N5‐oxide flavin species was proposed upon oxidation of a reduced flavin,^6^, ^17^, ^18^, ^19^, ^20^, ^21^ of which the structural and mechanistic delineations remain elusive. Given the reactive transient natures, structurally well‐defined flavin C4α/N5‐oxide adducts are noticeably rare in the Protein Data Bank. Hmo oxidase and monooxygenase‐like Y128F, nevertheless, lend an opportunity to allow one to physically visualize reactive C4α/N5‐oxide adducts and trace down their destinations. The information gained from this study should be very conducive to developments of new reactions and/or biocatalysts.

## 2 RESULTS AND DISCUSSION

### 2.1 *Trapping of carbanionic intermediates*

**FIGURE 1:**
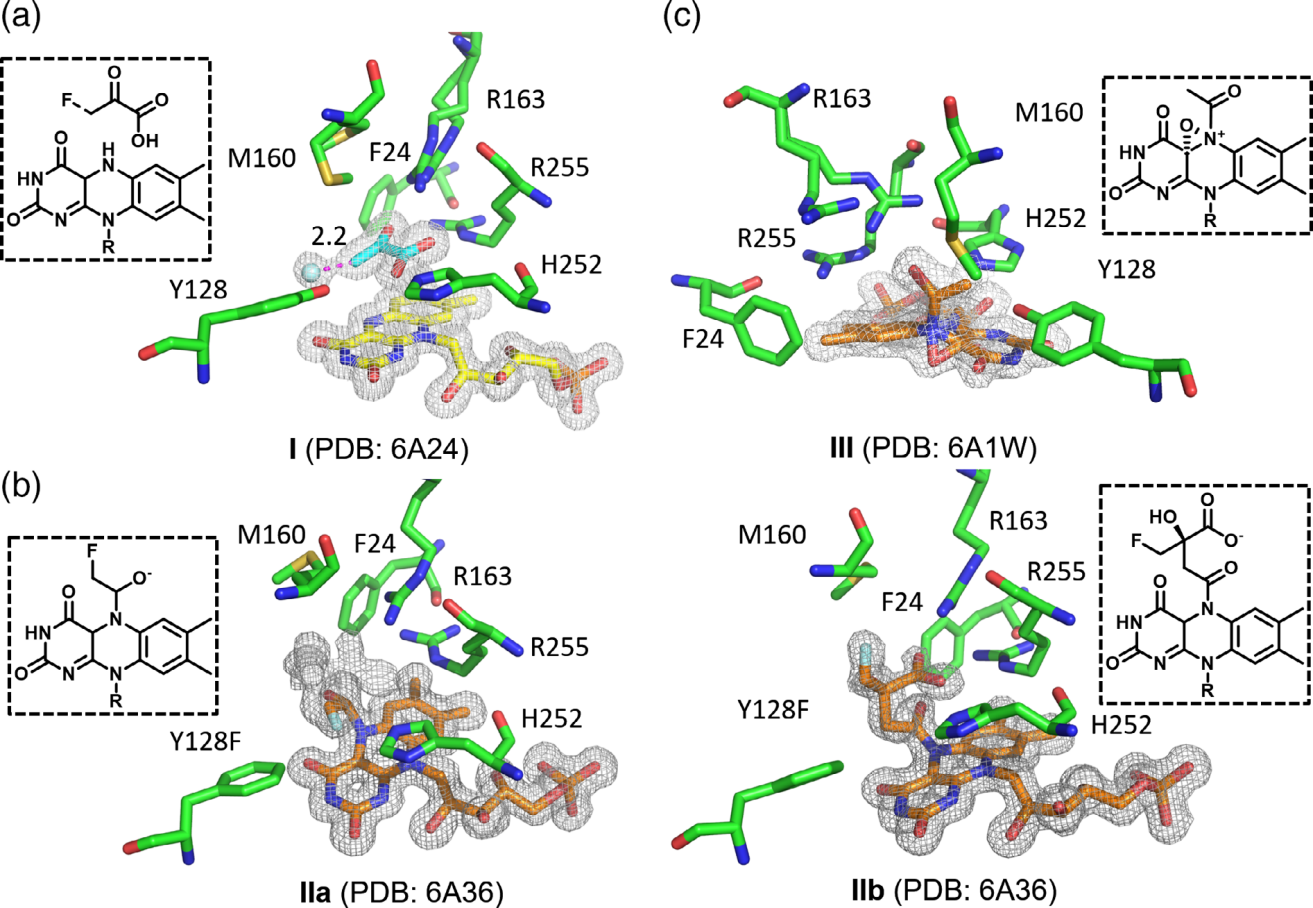
Trapping FMN‐peroxide anion using monofluoropyruvate. (a) The crystal structure of Hmo/Y128F in complex with 3‐monofluoropyruvate, where β‐fluorine is in a bond‐cleavage manner (2.2 Å) under the stereoelectronic effect (**I**). (b) The crystal structure of Y128F in complex with 3‐monofluoropyruvate, where 3‐monofluoropyruvate is associated with FMN to form double conformations, an N5‐alkanol‐C′α^−^‐C4α^+^‐FMN_ox_ zwitterion (**IIa**) or an N5‐γ‐fluoro‐β‐hydroxyl,β‐carboxyl‐butyryl‐FMN_ox_ conjugate (**IIb**). (c) The crystal structure of Hmo in complex with 3‐monofluoropyruvate, where β‐elimination and charge transfer coupling with molecular oxygen have taken place to form a C4α‐N5‐epoxide‐N5‐enoyl‐FMN_ox_ zwitterion (**III**)

**FIGURE 2:**
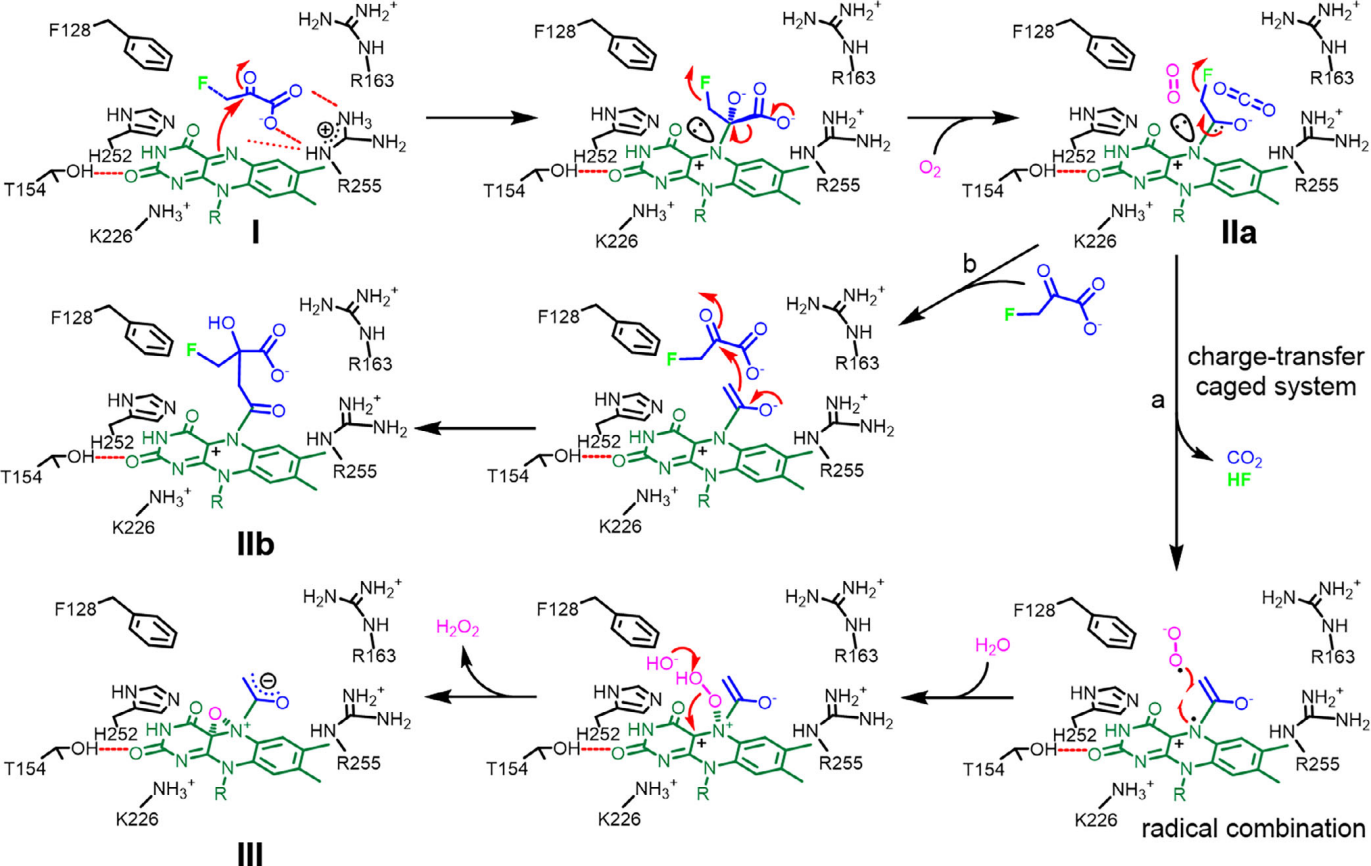
Proposed mechanism for the formation of complexes **IIa**, **IIb**, and **III** in Hmo/Y128F in the presence of 3‐monofluoropyruvate. The flavin adducts all are at the *si*‐face of the isoalloxazine ring. Free ligands, FMN, FMN adducts, and active‐site residues are colored cyan, yellow, orange, and green, respectively. The fluorine atom is colored green. Refer to Figures S5A, S5B, and S9 for stereo views and F_o_ − F_c_ omit difference electron density maps

Given the identification of N5‐acyl‐FMN_red_ adducts in Hmo or Y128F,^2^ the proposed intermediate N5‐benzoyl‐FMN_ox_‐C′α‐carbanion intermediate should exist, as upon decarboxylation the resulting lone pair should stay at C′α of an N5‐α‐hydroxyacid‐FMN_ox_ adduct (Scheme 1b). If the C′α‐carbanion can be physically or chemically trapped, FMN_ox_ that possesses nucleophilicity can be corroborated. This process is in analogy to that of thiamine diphosphate‐dependent decarboxylases,^22^, ^23^, ^24^, ^25^ where the decarboxylation of α‐ketoacid takes place by forming a covalent C2‐C′α adduct with the thiazolium ring of the thiamine diphosphate cofactor. To tackle this problem, β‐trifluoropyruvate was used to trap the C′α‐carbanion, whereby upon decarboxylation the resulting lone pair at C′α should be redirected away from assimilation into the FMN_ox_ electron sink by the strong electronegativity of the three β‐halogen atoms.^26^, ^27^ This attempt, however, failed, because α‐keto was unexpectedly hydrated forming an α‐gem‐diol species in all WT/Y128F crystals due to excessively strong electronegativity of the three β‐fluorine atoms (Figure S1a). β‐Difluorophenylpyruvate then was used to substitute β‐trifluoropyruvate. Despite a lesser extent of overall electronegativity, the α‐hydration effect still cannot be avoided (Figure S1b). β‐Monofluorophenyllactate (Figure S1c) or β‐monofluoropyruvate was thus used as the last measure, whereby the α‐hydration effect is minimized. Except an unwanted enantiomer (2*R*,3*S*)‐β‐fluorophenyllactate, three unprecedented FMN adducts were obtained when WT/Y128F crystals soaked with β‐monofluoropyruvate (two in WT and one in Y128F; Figure 1a–c): the first one harbors a free β‐monofluoropyruvate in the active site (in WT), where both the terminal carboxyl and α‐keto groups lie in the same plane. N5 of FMN_ox_ points toward C′α favorable for the formation of a C′α‐N5 linkage. Unexpectedly, the C—F bond is much longer (2.2 Å) than a normal C—F bond (1.35–1.39 Å), where the carboxyl group of β‐monofluoropyruvate is in alignment with the σ* antibonding orbital of C′β by a stereoelectronic effect, thus facilitating elimination of the β‐fluorine atom (**I**, Figure 1a). Interestingly, the carboxyl group of 3‐fluoro pyruvate is in close contact to both the imidazole ring (1.8 Å) of His252 and the guanidino group (2.6 Å) of Arg255 likely in strong H‐bond and electrostatic interactions, respectively. Given that both groups (carboxyl and imidazole) presume to be nucleophilic and that the imidazole ring remains planar (no addition reaction), the interaction probably is not in a covalent state. Based on the model, the planar 3‐fluoro pyruvate lies right above the isoalloxazine plane in a *π*–*π* stacking manner in 3.0 Å. The fluorine atom of 3‐fluoro pyruvate positioned in a dissociative manner is subject to both the strong electronegativity and the stereoelectronic effect. Considering the electronical conduction and induction ensemble amid 3‐fluoro pyruvate, isoalloxazine, and residues Arg255 and His252 in the spatially and temporally unique setting in a rigid and well‐packed crystal, the carboxyl group of 3‐fluoro pyruvate and the imidazole ring of His252 is brought to such an extraordinary short distance of 1.8 Å. The second one (in Y128F) presents two structural configurations, an N5‐alkanol‐C′α^−^‐C4α^+^‐FMN_ox_ zwitterion (**IIa**) and an N5‐γ‐fluoro‐β‐hydroxyl,β‐carboxyl‐butyryl‐FMN_ox_ conjugate (**IIb**), together confirming that decarboxylation takes place after formation of the N5–C′α bond (**IIa** and **IIb**, Figure 1b). **IIa** stands for a metastable zwitterion, where C′α is likely a metastable carbanion (because the β‐fluorine atom is still retained at C′β) and the tertiary C4α holds an sp^2^ configuration (carbocation). **IIb**, instead, is a condensation product, in that the C′α lone pair was attracted by β‐fluorine to form an enolate anion upon β‐elimination, which subsequently conjugates with a second molecule of β‐monofluoropyruvate by an aldol‐condensation reaction. The third one (in WT) represents a C4α‐N5‐epoxide‐N5‐enoyl‐FMN_ox_ adduct (**III**, Figure 1c), where the lone pair at C′α is hybridized with C′β π‐orbital resulting in an ethenol species in concert with β‐elimination. An additional electron density that bulges out the edge of C4α–N5 best fits an epoxide conformation. We reasoned that O_2_ in the active site is mediated by the lone pair at N5 through the single‐electron transfer and radical rebound mechanism to form an N5‐ethenol‐N5‐OO^−^ species in a way similar to EncM for an oxidative Favorskii‐type rearrangement.^18^ This peroxide species is off trajectory toward C′α thus unable to form the C′α‐peroxide. Instead, it may be quenched by H_2_O to form H_2_O_2_/H_2_O and N5‐O^−^. This N5‐O^−^ species is in a close proximity to the unoccupied orbital of C4α^+^ thereby bringing about the C4α–N5 epoxide at the *re*‐face of the flavin (Figure 2). The epoxidation of FMN requires a series of reactions, starting with ylide polarization, followed by α‐ketoacid‐FMN coupling, decarboxylation, β‐elimination, charge‐transfer rebound peroxidation, disproportionation, and finally ending up with epoxidation. Given the Y128F‐mediated oxidative decarboxylation reaction,^1^ the identified C′α carbanion is in an on‐course trajectory able to act on the distal O atom of C4α‐OOH. The collapse of C′α‐oxyanion would result in the C′α–N5 bond disassociation and formation of acetate, H_2_O and FMN_ox_, thus providing an alternative mechanism to the Baeyer–Villiger type reaction (Figure S2a). The identification of the N5–O^−^ species should facilitate identification of additional examples of flavin‐N5‐oxide‐utilizing oxygenases, although the essential reaction motif is yet to be established.

### 2.2 *Identification of C4α‐oxide intermediates*

**FIGURE 3:**
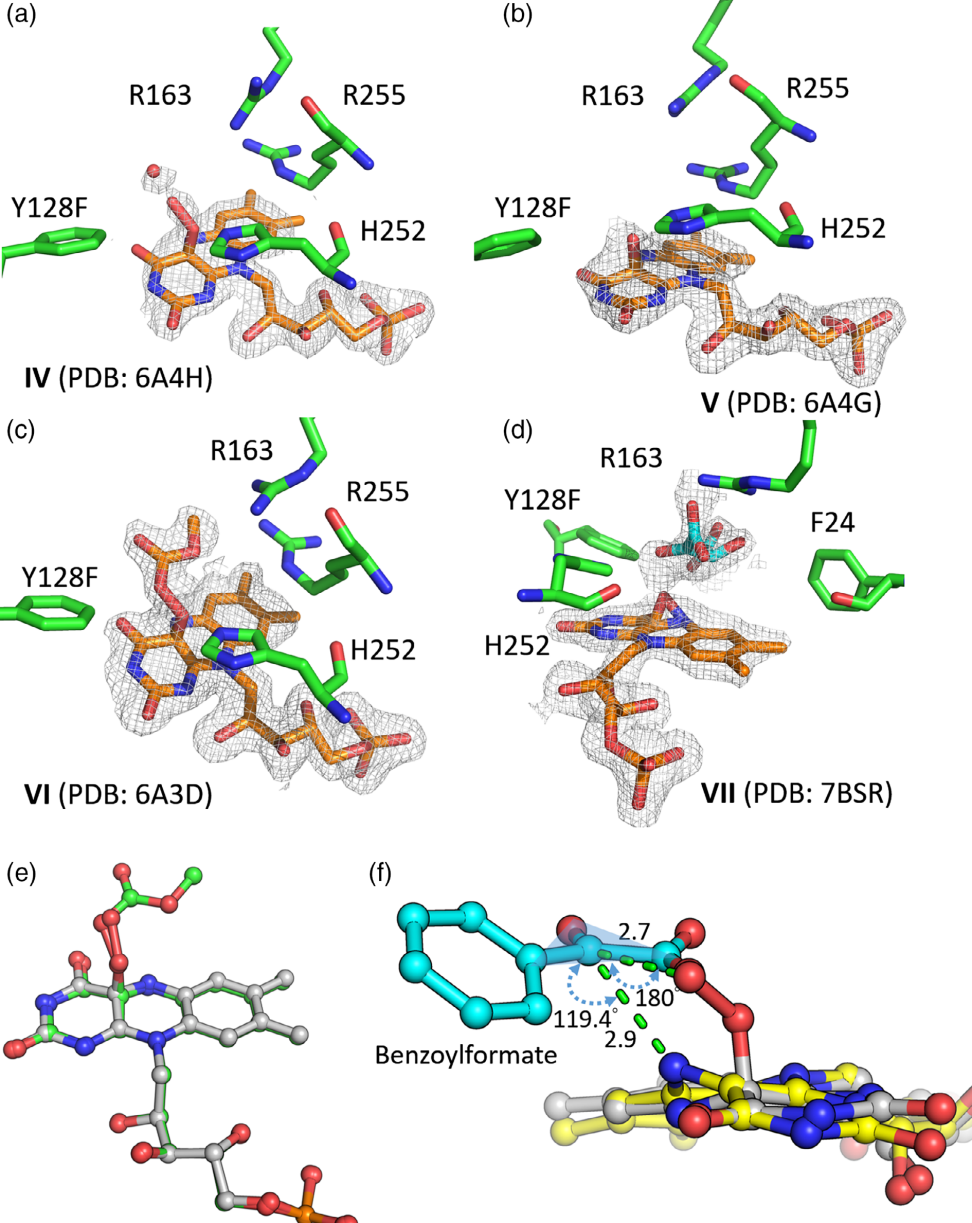
C4α‐oxide crystal structures. (a) The crystal complexes of C4α‐OO^−^‐FMN_red_ (**IV**, a peroxide species), (b) C4α‐OH‐FMN_red_ (**V**, a mono‐oxide species), (c) C4α‐OO‐methyl ester‐FMN_red_ (**VI**, a peroxide species), (d) 2‐hydroxy‐3‐oxosuccinate and C4α‐N5‐epoxide‐FMN_red_ (**VII**, an epoxide species) in Y128F. (e) Superposition of complexes **IV** (colored grey) and **VI** (colored green), where both peroxide moieties are well aligned. (f) The reaction trajectory, where the sp^3^‐hybrided N5 atom (colored yellow; 2.9 Å to Cα′) is positioned at an advantageous Bürgi–Dunitz angle (119.4°) as opposed to C4α‐OO^−^ (colored grey; 2.7 Å to Cα′), which is disfavorable in an attacking angle of 180° (in parallel with the α‐keto trigonal plane). The flavin adducts all are at the *si*‐face of the isoalloxazine ring. FMN adducts and active‐site residues are colored orange and green, respectively. The 2F_o_ − F_c_ electron density map is contoured at 2 σ. Refer to Figures S5C, S5D, S5E, S5F, and S10 for stereo views or views from different angles and F_o_ − F_c_ omit difference electron density maps

Given a flavin C4α‐peroxide, the distal oxygen atom of the peroxide species that serve as either a nucleophile or an electrophile is contingent on its protonation state. While the reactivity of the C4α‐oxide species has been well perceived, its visualization remains sporadic because of its instability and high reactivity. Orville et al. has nevertheless identified a flavin C4α‐OH moiety in a choline oxidase.^9^ The adduct was detected using a dedicated spectroscopic strategy for crystalline proteins, which is not a homogenous physical entity. Another recent report presented that a flavin ring (FAD) in a pyranose dehydrogenase PDH from *Agaricus meleagris* was transformed with a monooxide covalently linked to C4α at the *re*‐face of the isoalloxazine plane.^16^ With these encouraging findings, we were prompted to trap new reactive C4α‐oxide species, as the information should be important for the understanding of true capacities of flavoenzymes. Nonphysiological substrates methyl (*S*)‐mandelate (a nondecarboxylable analog) and tartaric acid (a poor substrate) were therefore employed to search for novel C4α‐oxide intermediates. The crystals of Y128F soaked with methyl (*S*)‐mandelate at different time intervals were subjected to X‐ray diffraction. By this practice, new C4α‐peroxide adducts, including C4α‐OH‐FMN_red_, C4α‐OO^−^‐FMN_red_, and C4α‐OO‐methyl ester‐FMN_red_ (Figure 3, **IV**–**VI**) at the *si*‐face of isoalloxazine ring, were obtained. In general, the identification of these complexes is in commensurate with resumption of Baeyer–Villiger‐type reactions. The reaction rationale is provided as follows: **IV** is formed in the first place upon 2‐electron oxidation of methyl (*S*)‐mandelate, because the C4α‐peroxide is relatively long‐lived in the absence of *p*‐OH (Y128F) and the methyl ester is not decarboxylable. The peroxide anion either undergoes self‐decay or is quenched by water (2.2 Å) to form **V**. Alternatively, the distal oxygen of C4α‐OO^−^ may attack the terminal methyl ester (instead of the expected α‐keto group) of an oxidized product, whereby benzaldehyde is freed and **VI** is formed upon collapse of the resulting oxyanion (Figure 5a).

### 2.3 *Stopped‐flow kinetics*

**FIGURE 4:**
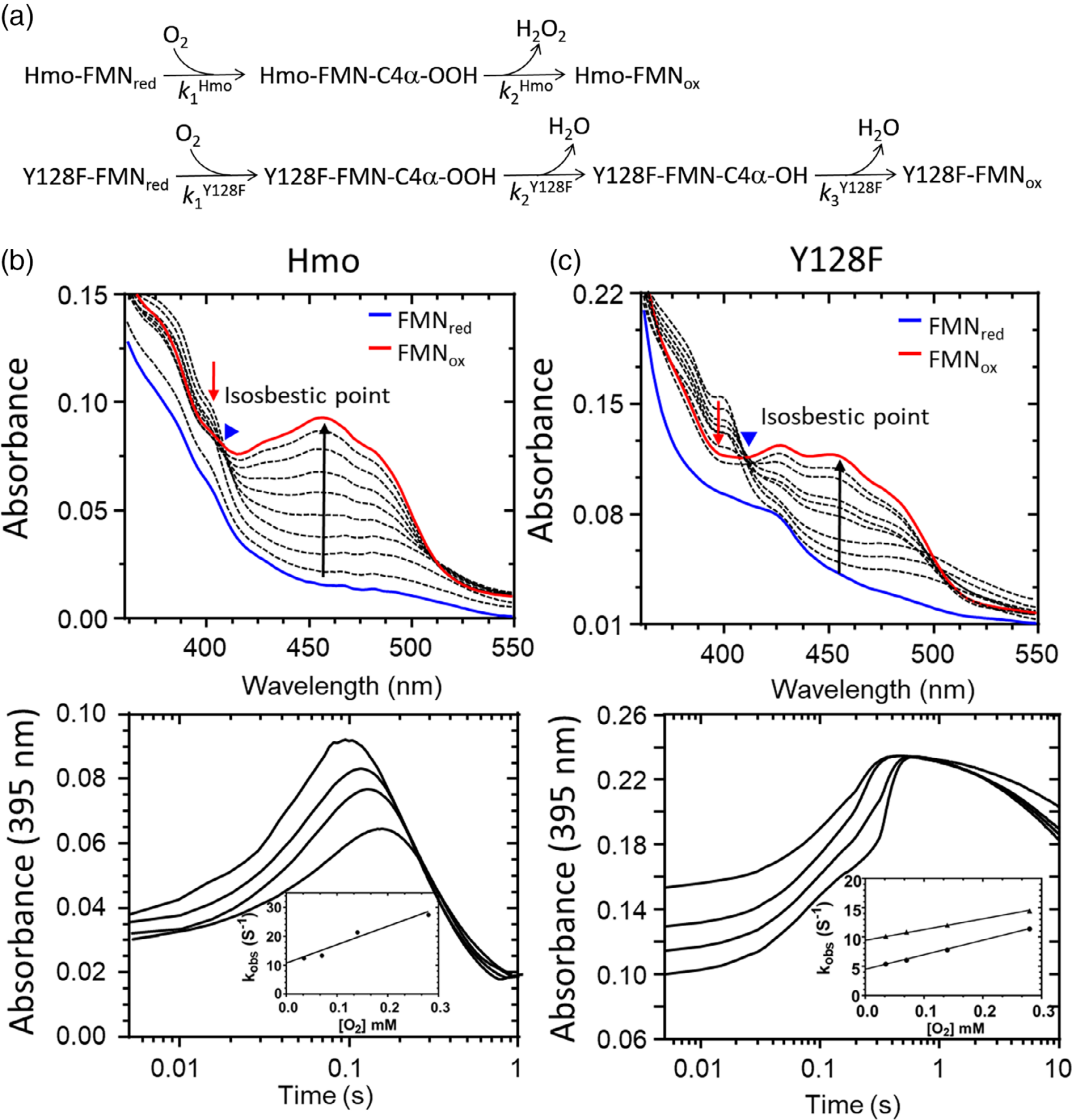
Formation of C4α‐Oxide catalyzed by Hmo and Y128F and kinetics thereof. (a) Proposed two‐ (upper path) and three‐phase (lower path) reactions for reduced Hmo and Y128F, respectively, in reaction with O_2_. (b), (c) UV‐Vis spectra (upper panel) recorded by a stopped‐flow‐PDA when reduced Hmo/Y128F (0.2 mM in 50 mM HEPES buffer, pH 7.0) was mixed with a O_2_‐containing buffer solution over a transient period of time (arrows indicate decreased or increased absorbances at 395 or 455 mm; blue triangle indicates the isosbestic point); kinetic spectra (lower panel) recorded by a stopped‐flow spectrometer for reduced Hmo/Y128F (0.2 mM in 50 mM anaerobic HEPES buffer, pH 7.0) when mixed with the same buffer solution containing various levels of O_2_ in the final reaction solutions; the inset shows a plot of *k*_obs_ for the formation of C4α‐OO(H)‐FMN monitored from traces at 395 nm versus various concentrations of O_2_

In contrast to general flavin‐dependent oxidases, the reactive C4α‐OO^−^ species in reduced Hmo or Y128F (FMN_red_) can be spectroscopically detected using a stopped‐flow‐PDA apparatus for reactions in an anaerobic solution against an aerobic buffer solution over a transient period of time.^11^, ^28^, ^29^ The emergence of a peak at 395 nm denotes the formation of an C4α‐OO^−^ adduct as opposed to the characteristic double absorbance peaks at 370 and 450 nm for fully oxidized Hmo/Y128F (FMN_ox_) (Figure 4b,c, top panel). Despite that the C4α‐OO^−^ adduct can be formed in both Hmo and Y128F, the half‐life is rather different, that is, the one in Y128F is longer than that in Hmo. This result is consistent with our structural/biochemical analysis, where the FMN_red_:O_2_ complex in Hmo is rapidly disassociated as H_2_O_2_ (Figure S3). Transient kinetics for both reduced Hmo and Y128F in reaction with O_2_ was further conducted using a stopped‐flow equipped with a monochromatic light source set at 395/455 nm (Figure 4b,c, bottom panel). The kinetic spectra suggest two‐ and three‐phasic reactions with Hmo and Y128F, respectively (Figure 4a). For Hmo, the first phase (0.005–0.1 s) is highlighted with increased absorbance at 395 nm representing rapid formation of C4α‐OO(H)‐FMN_red_. The second‐order rate constant (*k*_1_^Hmo^) of 6.4 ± 0.5 × 10^4^ M^−1^ s^−1^ was derived on the basis of the linear curve of *k*_obs_, which is proportional to O_2_ concentrations. The second phase (0.1–1 s), however, is independent of O_2_ concentration in correspondence to the elimination of H_2_O_2_ with a rate constant (*k*_2_^Hmo^) of 12.7 s^−1^ (Figure S7a). Both the bell‐shaped curve and fast secondary rate constants of Hmo are comparable to those of typical oxidases, such as pyranose 2‐oxidase (5.8 × 10^4^ M^−1^ s^−1^),^12^, ^29^ putrescine oxidase (4.3 × 10^4^ M^−1^ s^−1^),^30^ and dihydroorotate dehydrogenase (6.2 × 10^4^ M^−1^ s^−1^).^31^ For Y128F, the absorbance at 395 nm was similarly increased but lasted up to 1 s (0.005–1 s), roughly 10‐fold longer than that of Hmo. This unique pattern suggests that C4α‐OO(H)‐FMN_red_ in Y128F is more stable than its counterpart in Hmo. The upward absorbance curve can be further dissected into two sub‐phases in conformity with formation of C4α‐OO(H)‐FMN_red_ and C4α‐OH‐FMN_red_ at the first and second sub‐phases with the second‐order rate constants of 2.4 ± 0.6 × 10^4^ (*k*_1_^Y128F^) and 1.7 ± 0.3 × 10^4^ M^−1^ s^−1^ (*k*_2_^Y128F^) for each. The formation rate of C4α‐OO(H)‐FMN_red_ in Y128F is approximately 2.5‐fold slower than that in Hmo. The decay rate to C4α‐OH‐FMN_red_ at the second sub‐phase is somewhat slower than the peroxide formation rate at the first sub‐phase. FMN_ox_ is presumed to be reinstated after elimination of water from C4α‐OH‐FMN_red_, while this monoxide species is relatively stable (with a *k*_3_^Y128F^ rate constant of 7.2 s^−1^, Figure S7b) as the third phase sustains up to 10 s (1 ➔ 10 s). The three‐phased kinetics of Y128F, nevertheless, is analogs to some flavin‐dependent monooxygenases, such as L‐ornithine monooxygenase and *p*‐hydroxyphenylacetate hydroxylase (with rate constants of 0.021 and 0.35 s^−1^, respectively).^4^, ^32^

### 2.4 *Proposed mechanisms for formation of flavin‐oxide intermediates*

**FIGURE 5:**
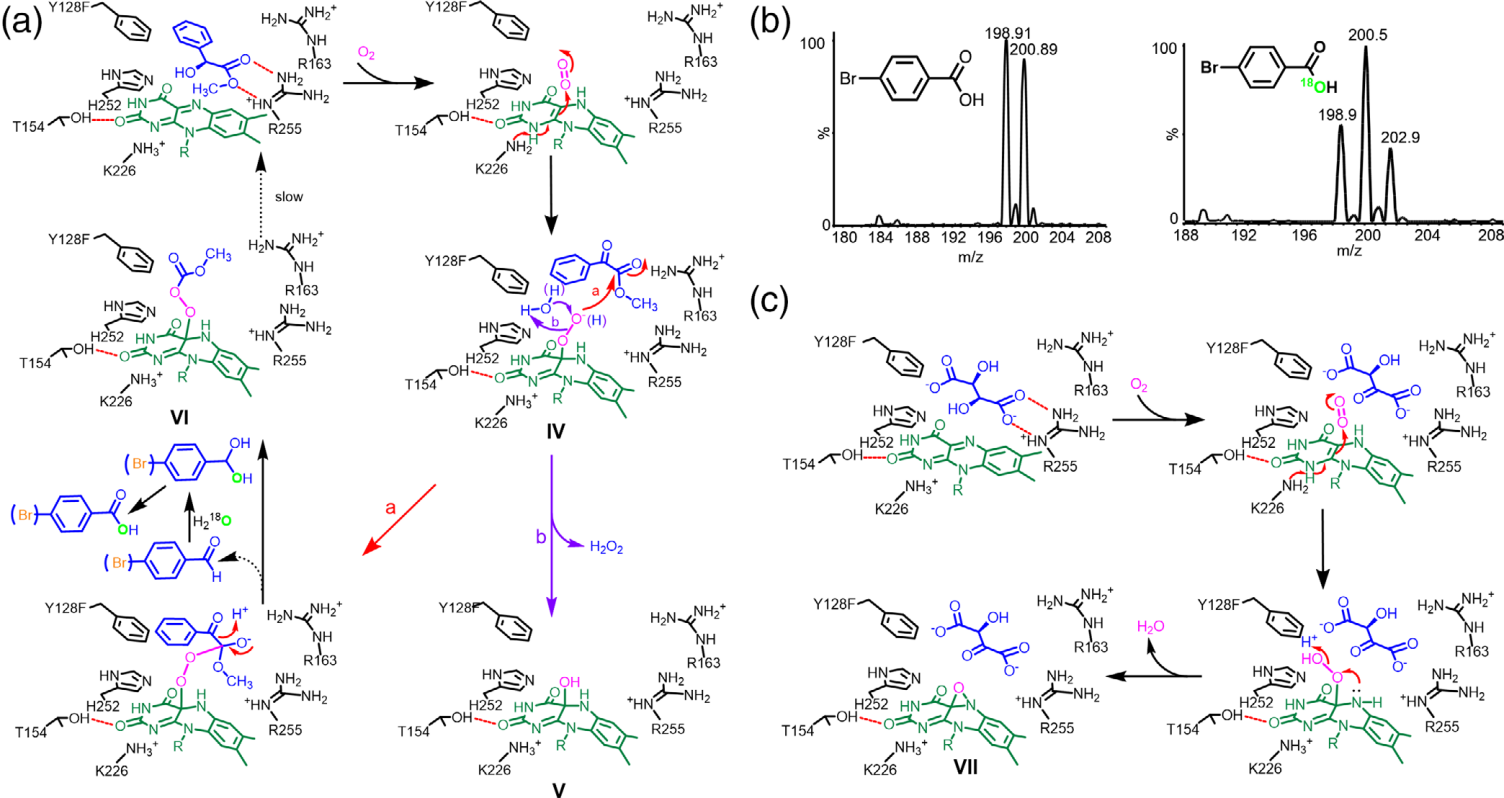
Proposed mechanisms for the formation of complexes **VI** and **VII**. (a) The proposed mechanism for the formation of complex **VI** by Y128F in the presence of methyl (*S*)‐mandelate or *p*‐bromo‐(*S*)‐mandelate. (b) The mass spectra of *p*‐bromobenzoate from the Y128F‐medaited reactions conducted in a H_2_O (left panel) or H_2_^18^O (right panel) buffer solution. (c) The proposed mechanism for the formation of complex **VII** by Y128F in the presence of 2*S*,3*R*‐tartrate

The kinetic analysis is appreciably in agreement with our structural observations. Since benzoic acid rather than benzaldehyde was detected in enzymatic reactions with methyl (*S*)‐mandelate as the substrate (Figure 5a), isotopic labelling experiments were further performed in a H_2_^18^O (80%) buffer solution using isotopically distinctive methyl *p*‐bromo‐(*S*)‐mandelate as the substrate. The reaction was quenched and subjected to LC–MS analysis in due course. The isotopic mass spectrum clearly showed that *p*‐bromo‐benzoate is incorporated with 50% ^18^O (M:M + 2:M + 4 = 1:2:1, Figures 5b and S6), thereby concluding that benzaldehyde is formed in the first place and undergoes hydration to allow a second oxidation reaction to proceed (Figure 5a). Superposition of **IV** over **VI** suggests that both peroxide moieties are well aligned, implicating that the terminal oxyanion of C4α‐peroxide is on‐track toward the terminal ester (Figure 3e). In contrast, given (*S*)‐mandelic acid the sp^3^ N5 lone pair (reduced FMN) is at preponderant Bürgi–Dunitz and Flippin–Lodge angles toward benzoylformate as opposed to C4α‐OO^−^, which is in parallel with the α‐keto trigonal plane at a dis‐favorable attacking angle (Figure 3f).

Because of a given substrate that will bring on a specific reaction in Y128F, we thus soaked the crystals with a higher concentration of tartaric acid, a poor substrate, in an attempt to slow down the reaction and see what would result. Surprisingly, an epoxide adduct (N5‐C4α‐epoxide‐FMN_red_) instead of a peroxide one was formed in crystals (Figure 3d, **VII**), reinforcing that the substrate is a pivotal factor and the *p*‐OH group is intrinsically involved in leverage of the oxidation cascade. For the formation of the epoxide adduct our reasoning is that: One molecule of tartaric acid (the 2*S*,3*R*‐enantiomer) undergoes oxidation to form 2‐hydroxy‐3‐oxosuccinate with concomitant reduction of flavin (FMN_red_). Unlike benzoylformate the resulting product did not flip in the active site likely due to its bulky and charged groups. The FMN_red_ then reacts with squeezed‐in O_2_ to form a peroxide species. Because of the product in a dis‐favorable orientation (with α‐keto pointing toward His252), both N5 and C4α‐OO^−^ cannot follow the typical route of a Baeyer–Villiger‐type reaction. Instead, the N5 lone pair attacks the proximal O atom of C4α‐OO^−^ to form N5‐C4α‐epoxide‐FMN_red_, where the distal O is discharged as H_2_O. The epoxide is placed above the *si* face of isoalloxazine as opposed to the C4α‐N5‐epoxide‐N5‐enoyl‐FMN_ox_ adduct (**III**), of which the epoxide is positioned at the *re* face of isoalloxazine (Figure 5c). In this context (the unique spatial position and orientation of the substrate), C4α‐OO^−^ is no longer a nucleophile but instead is poised to be acted on by N5 to form an epoxide. To this end, both the chemical property and the geographic orientation of a given substrate in the active center are intrinsic effectors, which lead to a specific reaction destination.

## 3 CONCLUSION

As described above, when un‐decarboxylable methyl ester was used as a substrate, the formation of the N5‐Cα′ linkage would be temporary as it is in a futile cycle of association/disassociation. In contrast, the superimposition of N5‐phenylacetyl‐FMN and C4α‐OO‐FMN shows that the peroxide moiety is sterically compatible with the N5‐phenylacetyl moiety for the bond formation between C4α‐OO^−^ and Cα′‐carbanion (as a result of decarboxylation; Figure S8). Therefore, the reaction tendency for either oxygenation or the Baeyer–Villiger‐type or the peroxide‐decarboxylation‐assisted reaction is dependent on the ensemble of the attacking trajectory, binding geometry, and physiochemical property of a reactant in the active milieu. Given that lactate monooxygenase (LMO) preserves the active‐site tyrosine residue (equivalent to Y128) and carries out the 4‐electron oxidation reaction (lactate to acetate), LMO (with *p*‐OH) likely follows the Baeyer–Villiger‐type (Figure S2Ba) or peroxide‐decarboxylation‐assisted (Figure S2Bb) oxidative decarboxylation reaction under the spatiotemporal constraint in LMO, while Y128F (without *p*‐OH) may favor the peroxide‐N5‐assisted oxidative decarboxylation reaction or permit both types of reactions (Figure S2a).^18^ While flavoproteins that are omnipresent in all living organisms have been studied over a century, many issues remain outstanding. In this study, structural systems biology in conjunction with synthetic probes allows us to witness some reactions, which were unknowingly omitted previously. Given the Hammond postulate,^33^ the transition state resembles a diradical species, of which the extent of the perturbation dictates the oxidation to follow the one‐ or two‐electron mechanism. Provided monofluoropyruvate, three transition‐like complexes **I**, **II**, and **III** identified here fulfill the theory. Four long‐sought C4α‐oxide adducts (**IV**–**VII**), on the other hand, back the monooxygenation, Baeyer–Villiger/peroxide‐decarboxylation assisted reactions for Y128F as a monooxygenase. In addition to the physiochemical properties of a given substrate, the reaction propensity further depends on the extent of nucleophilicity of the peroxide, which is compromised in Hmo but enhanced in Y128F. Our results thus establish that FMN‐dependent enzymes are multifaceted, where the effectiveness of the flavin cofactor is governed by both the unique active‐site motif of a given enzyme and the physicochemical property of a selected ligand. These findings should be very informative for development of new biocatalysts or chemical reactions.

## 4 MATERIALS AND METHODS

### 4.1 *Expression and purification of Hmo and Y128F*

The gene that encodes *hmo* (*orf22*) was amplified from *Amycolatopsis orientalis* genomic DNAs and expressed as an N‐terminal His_6_‐tagged protein using the expression vector pET‐28a (+). Briefly, the ligated plasmids of native *hmo* and mutant Y128F were transformed into *Escherichia coli* BL21(DE3) cells and cultured in 1 L of LB medium containing 50 mg L^−1^ kanamycin at 37°C until A_600_ 0.6. For protein expression and purification, 200 μl of 1.0 M isopropoyl‐*β*‐d‐thio‐galatoside (IPTG) was added in cultured *E. coli* medium for further 12 hr at 16°C. After cell lysis and clarification by centrifugation in binding buffer (20 mM HEPES at pH 7.5, 500 mM NaCl, 10 mM imidazole, and 10% glycerol), the supernatant liquid was applied onto an Ni^2+^‐NTA resin column for further purification. The column was washed twice with buffers in different concentrations (first wash: 20 mM HEPES at pH 8.0, 500 mM NaCl and 40 mM imidazole; second wash: 20 mM HEPES at pH 8.0, 500 mM NaCl and 80 mM imidazole) before eluting the target protein with 15 ml of the elution buffer (20 mM HEPES at pH 8, 500 mM NaCl, 250 mM imidazole). All proteins were further purified by size‐exclusion chromatography on an Äkta FPLC system equipped with a HiLoad 16/60 Superdex‐200 column under an isocratic condition (20 mM HEPES, pH 8.0, 100 mM NaCl). Protein purity and concentration were examined by SDS‐PAGE and the Bradford assay with BSA as a standard.

### 4.2 *Crystallization and data collection*

Hmo and mutant Y128F were crystallized using a hanging drop vapor diffusion method at 20°C by mixing the protein with the reservoir solution consisting of 35% Tascimate, 0.1 M Bis‐Tris propane, pH 7.0. Crystals were generally formed after a 5‐day incubation. Before X‐ray diffraction data collection, crystals were transferred to the same mother liquor containing 20% glycerol as a cryoprotectant. For complexed structures (the Hmo/mutants crystals soaked with trifluoropyruvate, β‐difluoro‐phenylpyruvate, β‐monofluoro‐phenylpyruvate, or β‐monofluoropyruvate), X‐ray diffraction data sets were recorded after soaking the protein crystals with 35 mM of a given substrate for 10 min up to 24 hr in the reservoir solution. All crystal diffractions were collected at the National Synchrotron Radiation Research Center (NSRRC) (Taiwan) beamline 13B1, 13C1, 15A1, and 05A (equipped with an ADSC Quantum‐315 or MX300HE CCD detector in an operating temperature of 100 K) or at beamline 44XU of Spring‐8 (Japan). Data were indexed and scaled using the HKL2000 package.^34^ These crystals all belong to the I422 space group.

### 4.3 *Structure determination and refinement*

The crystal structures of mandelate oxidase and its mutants were determined by molecular replacement (MR) with Phaser‐MR from the CCP4 program suit.^35^ The crystal structure 3SGZ (Protein Data Bank [PDB] accession code) was used as the search model for solving the initial phase. The protein structures were built and refined by using REFMAC.^36^ Subsequent iterative cycles of model building and refinement were performed using COOT,^37^ PHENIX.^38^ Detail refinement statistics are presented in Table S1. Both protein structures and electron density maps were generated using PyMOL.^39^

### 4.4 *Halogenated substrates synthesis and characterization*

Two commercially unavailable chemicals 3,3‐difluoro‐2‐hydroxy‐3‐phenylpropionic acid and 3‐fluoro‐2‐hydroxy‐3‐phenylpropanoic acid were chemically synthesized for liganded crystal structures and enzymatic assays. The synthetic procedures are detailed in Supporting Information. These two compounds were purified using column chromatography and characterized by MS or NMR. The ^1^H and ^13^C NMR spectra of these compounds were dissolved in DMSO‐*d*_6_ or CD_3_OD unless otherwise specified and acquired from Bruker Avance 600 spectrometers equipped with CryoProbe™. The chemical shifts of ^1^H NMR spectra were reported in units of ppm. The TopSpin (version 3.5) program was used to process NMR data sets and the detailed NMR spectra can be found in Figures S11 and S12.

### 4.5 *Stopped‐flow kinetics for reduced Hmo/Y128F in reaction with dioxygen*

All reactions of reduced Hmo/Y128F against various concentrations of O_2_ were carried out by using an Applied Photophysics stopped‐flow spectrometer SX20 in the single mixing mode. Before starting the experiments, the flow paths of the stopped‐flow apparatus were purged to be anaerobic by flushing with a deoxygenated buffer solution containing 0.5 mg/ml of dithionite in 50 mM HEPES (pH 7.0). Different quantities of O_2_ in solutions were prepared by purging with a desired ratio of O_2_ and N_2_ mixed gases to the buffer in an air‐tight bottle for 10 min, and measured the actual O_2_ concentration by oxygen electrode probe. For the sequential spectroscopic studies of reduced Hmo/Y128F with O_2_, the anaerobic enzyme solution was prepared by purging with helium in a gas‐tight bottle for several times and refresh with deoxygenated HEPES buffer solution containing dithionite (1.5 mg/ml) as the reductant to reach a final concentration of 0.2 mM. After mixing with the O_2_‐saturated buffer solution (final concentration: 0.28 mM), the UV‐vis spectra were recorded at wavelengths from 350 to 550 nm for 10 s by the stopped‐flow spectrophotometer with a photodiode array detector (PDA). For kinetic measurements of the C4α‐hydroperoxide formation, all reactions were carried out in the same buffer system and enzyme amount as the sequential spectroscopic studies, unless otherwise stated. The absorbance was recorded for the wavelengths set at 395 and 458 nm for detection of C4α‐OO‐FMN and FMN_ox_ in the oxidation of reduced Hmo and Y128F by O_2_ during the course of reactions. Rate constants from the kinetic traces of the enzymatic reaction were calculated by using Marquardt–Levenberg nonlinear fitting algorithms in ProKIV (Applied Photophysics software). The values of observed rate constant (*k*_obs_) calculated are 12.4, 13.3, 21.4, 27.5 and 5.4, 6.1, 7.8, 11.4 s^−1^ on the basis of reduced Hmo/Y128F in reaction with O_2_ at given concentrations of 0.035, 0.07, 0.14, and 0.28 mM.

### 4.6 *Enzymatic reactions of Y128F mutant conducted in H_2_^18^O*

The isotope labelling experiment was carried out in a buffer solution contains 90% H_2_^18^O (HEPES 20 mM, NaCl 100 mM, pH 7.5), substrates and the Y128F mutant at 25°C for 1 hr. After quenching with 6 N HCl, the filtered reaction solutions were subjected to LC–MS with a 4.6 × 250 mm, 5 μ Phenomenex C18 Prodigy C18 column mounted on the Waters Alliance 2695 HPLC module with a Xevo TQ‐S micro triple quadrupole mass spectrometry. The mobile phase was set as: water with 1% formic acid in Solvent A and acetonitrile with 1% formic acid in Solvent B, a linear gradient from 2 to 40% of Solvent B in Solvent A at a flow rate of 1.0 ml min^−1^ over 37 min followed by 98% of Solvent B for another 3 min. The analytes were monitored under a UV wavelength at 280 nm using both positive and negative modes for mass detection. Data was analyzed with the MassLynx software.

### 4.7 *PDB accession codes*

The atomic coordinates have been deposited in the Protein Data Bank with the accession codes 5ZZT, 6A24, 6A1W, 6A01, 6A1N, 6A1B, 6A0B, 6A36, 6A4H, 6A4G, 6A3D, and 7BSR.

## AUTHOR CONTRIBUTIONS

**Kuan‐Hung Lin:** Data curation; formal analysis; methodology. **Syue‐Yi Lyu:** Data curation; formal analysis. **Hsien‐Wei Yeh:** Data curation; formal analysis. **Yi‐Shan Li:** Data curation; formal analysis. **Ning‐Shian Hsu:** Data curation; formal analysis; investigation. **Chun‐Man Huang:** Data curation; formal analysis. **Yung‐Lin Wang:** Formal analysis; investigation. **Hao‐Wei Shih:** Investigation; methodology. **Zhe‐Chong Wang:** Investigation; methodology. **Chang‐Jer Wu :** Resources. **Tsung‐Lin Li:** Funding acquisition; supervision; writing‐original draft; writing‐review and editing.

## Supporting information


**Appendix** **S1.** Supporting Information
