# Supplementary material for: Structural and chemical trapping of flavin‐oxide intermediates reveals substrate‐directed reaction multiplicity
Source: Protein Sci. 2020 May 26;29(7):1655–66. doi: 10.1002/pro.3879 (PMC7314388; doi:10.1002/pro.3879)
Supplement: Supplementary file 1 — Appendix S1. Supporting Information [file PRO-29-1655-s001.docx]

**Supporting Information**

**Structural and chemical trapping of flavin-oxide intermediates reveals substrate-directed reaction multiplicity**

Kuan-Hung Lin,^‡ 1,2^ Syue-Yi Lyu,^‡ 1^ Hsien-Wei Yeh,^1^ Yi-Shan Li,^1^ Ning-Shian Hsu,^1^ Chun-Man Huang,^1^ Yung-Lin Wang,^1^ Hao-Wei Shih,^1^ Zhe-Chong Wang,^1^ Chang-Jer Wu ^3^ and Tsung-Lin Li *^1,3,4^

^1^ Genomics Research Center, Academia Sinica, Taipei 115, Taiwan.

^2^ The Institute of Biochemistry and Molecular Biology, National Yang-Ming

University, Taipei 112, Taiwan.

^3^ Department of Food Science, National Taiwan Ocean University, Keelung 202,

Taiwan

^4^ Biotechnology Center, National Chung Hsing University, Taichung City, 402, Taiwan.

^‡^ These authors contributed equally

^*^ Correspondence: [tlli@gate.sinica.edu.tw](mailto:tlli@gate.sinica.edu.tw)

**This Supplementary file includes:**

Materials and Methods

Figures S1 to S13

Tables S1

Scheme 1-2

References 1

**Materials and Methods**

**Compound characterization and synthesis**

Halogenated substrates for the crystal soaking and enzymatic assays were chemically synthesized, the detail procedures were presented as below. All compounds were purified using column chromatography and characterized by MS or NMR unless otherwise stated. ^1^H and ^13^C NMR spectra in DMSO-d_6_ or CD_3_OD for selected compounds were acquired from Bruker Avance 600 spectrometers equipped with CryoProbe^TM^, and chemical shifts of ^1^H NMR spectra were reported in units of ppm relative to tetramethylsilane (TMS). The TopSpin (version 3.5) program was used to process NMR data sets and detailed peak information (chemical shift δ; peak multiplicity; coupling constant *J*; proton number) was presented as follows: (refer to Figures S12-S13 for NMR spectra):

1. **Synthesis of 3,3-difluoro-2-hydroxy-3-phenylpropionic acid**The synthetic procedure for 3,3-difluoro-2-hydroxy-3-phenylpropionic acid is outlined in **Scheme 1**, in which benzoylformic acid was used as the starting material.

   **Scheme 1.**

**a. Synthesis of methyl benzoylformate (2)**

Methyl benzoylformate was prepared by mixing concentrated sulfuric acid with benzoylformic acid (**1**, 3 g, 20 mmol) in anhydrous methanol. The acidified aqueous phase was extracted with diethyl ether (3 × 25 mL). The organic layers were combined, washed with brine (3 × 25 mL), dried and evaporated to afford an oil. The crude extract was purified by column chromatography (EtOAc:hexane = 1:9 (v/v), 90% overall yield) to obtain compound **2**. LC-MS: m/z 165.14 [M+H]^+^.

**b. Synthesis of methyl difluorophenylethanoate (3)**

Methyl benzoylformate (**2**; 3.3 g, 20 mmol) were fluorinated by 0.25 molar excess of DAST. Reaction mixtures were gradually dissolved in CH_2_Cl_2_ with stirring for 5 hrs at 25°C; compound **3** was purified by column chromatography (EtOAc:hexane = 1:9 (v/v), 75% overall yield).

**c. Synthesis of 2,2-difluoro-1-methoxy-2-phenylethanol (4)**

To synthesize compound **4**, a solution of methyl difluorophenylethanoate (**3**, 2.79 g, 15.0 mmol), which was dissolved in MeOH (20 mL), was added with NaBH_3_ (624 mg, 16.5 mmol) at a -50°C cold bath. After 6 hrs of vigorous stirring, the slurry was quenched by a slow addition of 35 mL of 1 N HCl (at -50°C) and warmed back to room temperature. Diethyl ether (3 × 25 mL) was used to extract the organic phase. The reaction mixture was washed with water (2 × 25 mL) and dried to afford crude 2,2-difluoro-1-methoxy-2-phenylethanol (**4**), which was used in the next step without further purification.

**d. Synthesis of 3,3-difluoro-2-hydroxy-3-phenylpropionitrile (5)**

Crude compound **4** was dissolved in water with KCN (977 mg, 15.0 mmol) and KH_2_PO_4_ (2.04 g, 15.0 mmol). After vigorous stirring at room temperature for 5 hrs, the mixture was extracted with diethyl ether (3 × 25 mL) and washed sequentially with 2.0% H_2_S0_4_ and H_2_O (2 × 25 mL of each) before drying with a rotary evaporator. Dried residues were purified by column chromatography (EtOAc:hexane = 1:1 (v/v)), which afforded compound **5** about 2.0 g.

**e. Synthesis of ethyl 3,3-difluoro-2-hydroxy-3-phenylpropionate (6)**

Gaseous hydrogen chloride was slowly bubbled into a mixture of 3,3-difluoro-2-hydroxy-3-phenylpropionitrile (**5**; 2.0 g, 8.5 mmol) and absolute ethanol (2 mL, 1.57 g, 34 mmol) for 12 hrs at 25 °C. After that, 15 mL of deionized water was added into the reaction until it became clear. The solution was extracted with diethyl ether (4 × 25 mL) and followed by washing (2 × 25 mL of brine) and evaporation under reduced pressure. The organic layers were purified by column chromatography (EtOAc: hexane = 1:1 (v/v), 85% overall yield). LC-MS: m/z 231.21 [M+H]^+^.

**f. Synthesis of 3,3-difluoro-2-hydroxy-3-phenylpropionic acid (7)**

To obtain the final product, compound (**6**) (1.7 g, 7.2 mmol) was dissolved in 50% aqueous isopropanol (20 mL) with addition of NaHCO_3_ (1.26 g, 15 mmol), and heated to 50°C for 24 hrs. The solvents were sequentially evaporated, taken up in water (10 mL), washed with diethyl ether and acidified with 1.0 M HCl (15 mL). The acidified aqueous phase was extracted with diethyl ether (3 × 50 mL). After evaporation, compound **7** was purified by column chromatography (EtOAc:hexane = 1:1 (v/v)). LC-MS: m/z 201.10 [M-H]^-^. ^1^H-NMR of compound (**7**) (600 MHz, CD_3_OD): δ 4.58 (*dd*, J = 7.8, 4.8, 1H), 7.47 (*m*, 3H), 7.55 (*d*, J = 6.6, 2H); ^13^C NMR of compound (**7**) (600 MHz, CD_3_OD): δ74.9 (C2), 119.4 (C3), 127.3 (C5, C9), 129.3 (C6, C7, C8), 135.8 (C4), 171.9 (C1) (Figure S12)

1. **Synthesis of** **3-fluoro-2-hydroxy-3-phenylpropanoic acid**The synthetic procedure for β-fluoro-α-oxobenzenepropanoic acid is outlined in **Scheme 2**, in which the commercially available compound ethyl 3-phenylglycidate was used as the starting material.

**Scheme 2.**

**a. Synthesis of ethyl 3-fluoro-2-hydroxy-3-phenylpropanoate (2)**

Ethyl 3-phenylglycidate (**1**; 2.2 g, 11 mmol) was prepared by mixing with hydrofluoric acid in pyridine. The mixture was sequentially extracted with diethyl ether (3 × 20 mL), washed with brine (3 × 25 mL), dried and evaporated. The crude extract was purified by column chromatography (EtOAc:hexane = 1:9 (v/v), 65% overall yield) to obtain compound **2**. LC-MS: m/z 405.25 [M+H]^+^.

**b. Synthesis of 3-fluoro-2-hydroxy-3-phenylpropanoic acid (3)**

Ethyl 3-fluoro-2-hydroxy-3-phenylpropanoate (**2**; 1.5 g, 3.7 mmol) was dissolved in 90% EtOH (20 mL) and added with NaOH (10 mmol). The solution was stirred at 50°C for 24 hrs. Water-insoluble substances were extracted with diethyl ether (4 × 25 mL). After drying and evaporation, oil-type crude compounds resulted was subjected to column chromatography (EtOAc:hexane = 1:1 (v/v), 70% overall yield). LC-MS: m/z 185.17 [M+H]^+^. ^1^H-NMR of compound (**3**) (600 MHz, CD3OD): δ 4.100 (*q*, J = 7.2, 2H), 7.501 (*m*, 3H), 7.601 (*d*, J = 7.8, 2H) ; ^13^C NMR of compound (**3**) (600 MHz, DMSO-d^6^): δ 88.2 (C2), 89.4 (C3), 127.0 (C5, C9), 128.9 (C7, C8), 129.5 (C6), 135.1 (C4), 169.8 (C1) (Figure S13)

**Figures**

**A**


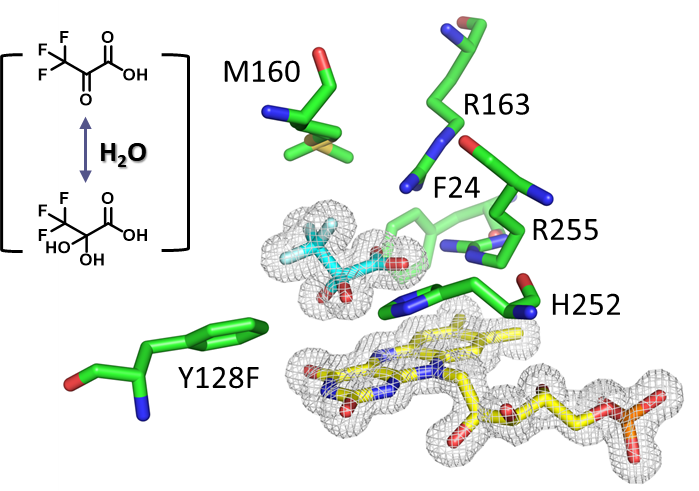


**B**

**
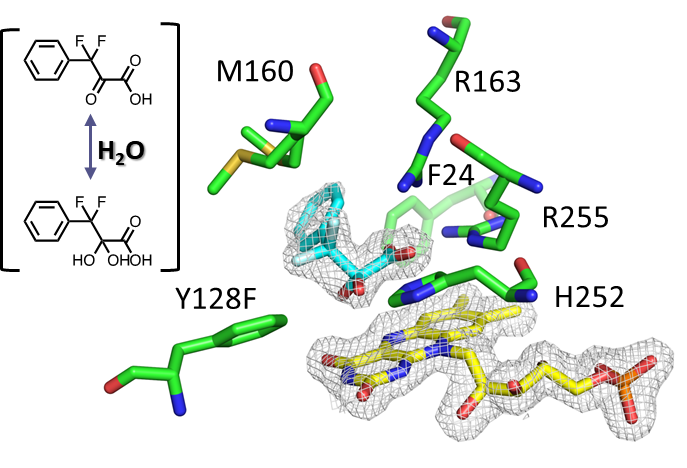
**

**C**


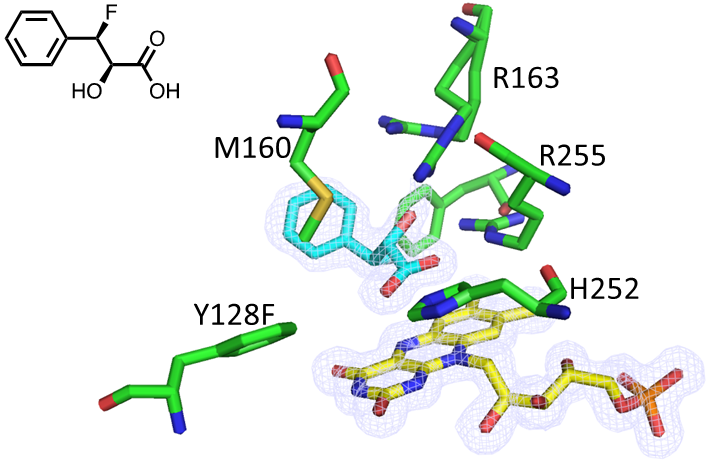


**Figure S1**. **Trapping FMN-adducts using fluorophenyl lactate/fluoropyruvate**. (A) The crystal structure of Y128F in complex with 3,3,3-trifluoropyruvate, where α-keto is hydrated to α-diol due to the strong electronegativity of β-fluorine atoms. (B) The crystal structure of Y128F in complex with 3,3-difluoro-3-phenylpyruvate, which experiences the same hydration effect. (C) The crystal structure of Y128F in complex with racemic 3-fluoro-3-phenyllactate, where only is (2*R*,3*S*)-3-fluoro-3-phenyllactate out the enantiomeric mixture retained in the active site. The 2F_o_-F_c_ electron density map is contoured at 2 σ.

**A.**

**B.**

**Figure S2**. **The mechanisms of oxidative decarboxylation catalyzed by Y128F and LMO**. (A) The proposed 4-electron oxidation catalyzed by Y128F, where the reaction follows the peroxide-N5-assisted decarboxylation disproportionation reaction to form benzoate. (B) The proposed 4-electron oxidation catalyzed by LMO (using Hmo numbering system for LMO), where the reaction follows the Baeyer-Villiger-type (Ba) or peroxide-decarboxylation-assisted (Bb) oxidative decarboxylation to form acetate.

**
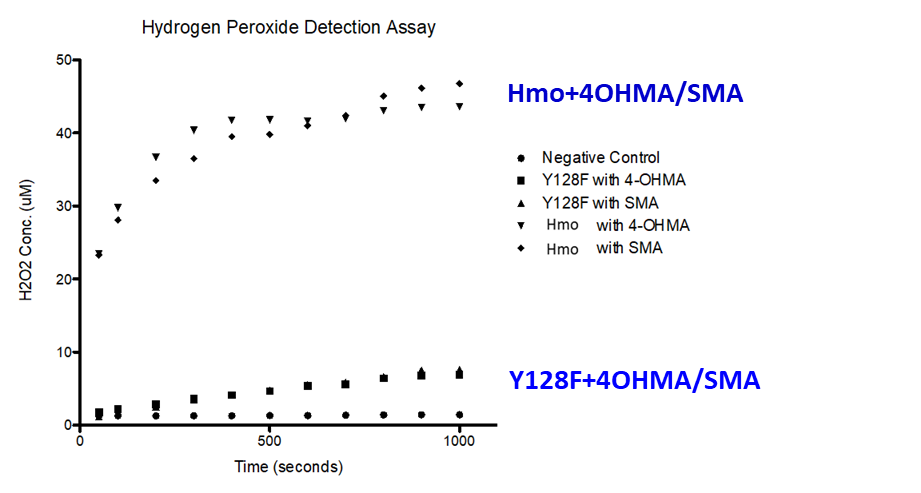
**

**Figure S3. Formation of hydrogen peroxide catalyzed by WT/Y128F in the presence of (*S*)-mandelate/4-OH-(*S*)-madelate.** The level of H_2_O_2_ in Y128F reactions is inverse to the level of WT reactions which suggests H_2_O_2_ is not free in solution but may be engaged in the oxidative decarboxylation reaction^[^[^1^](#_ENREF_1)^]^.

**
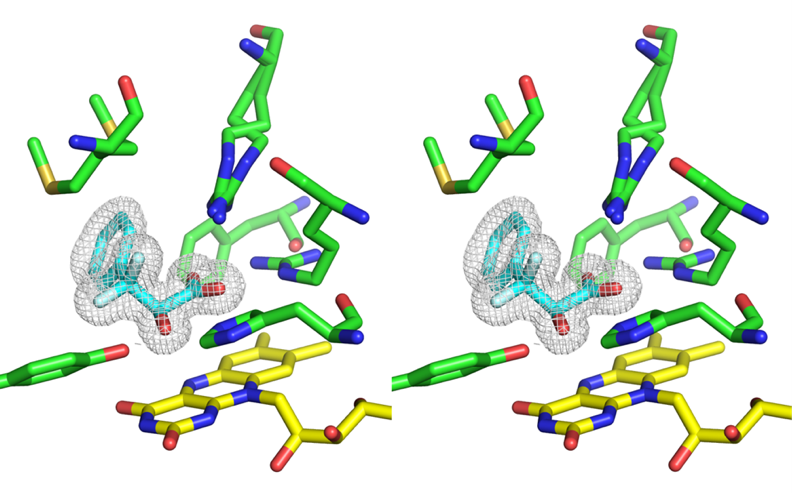
**

**PDB ID: 5ZZT**

**B.**

**
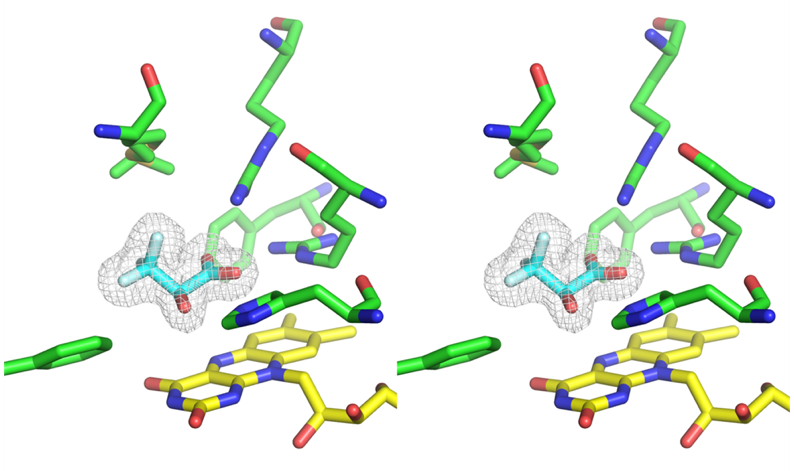
**

**PDB ID: 6A0B**

**C.**


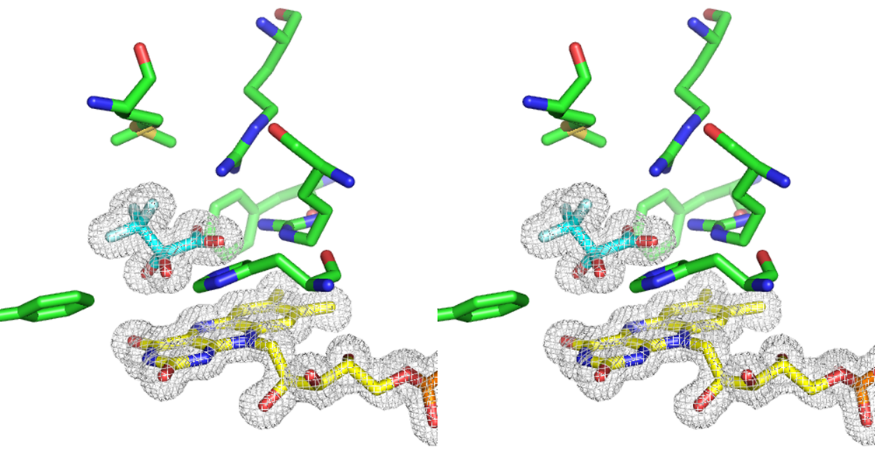


**PDB ID: 6A1B**

**D.**


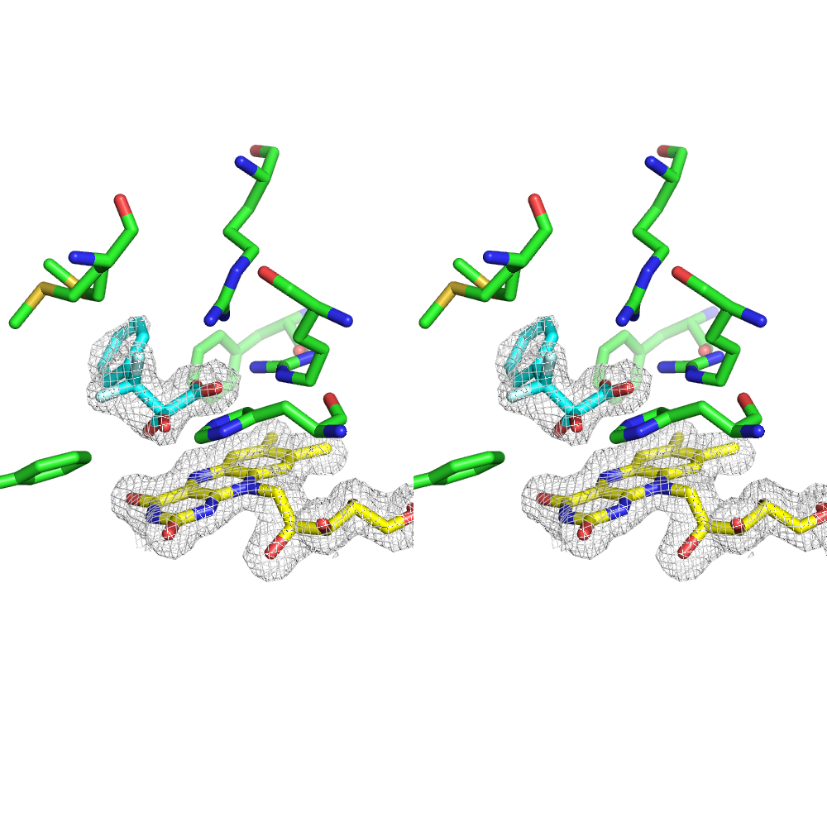


**PDB ID: 6A01**

**E.**


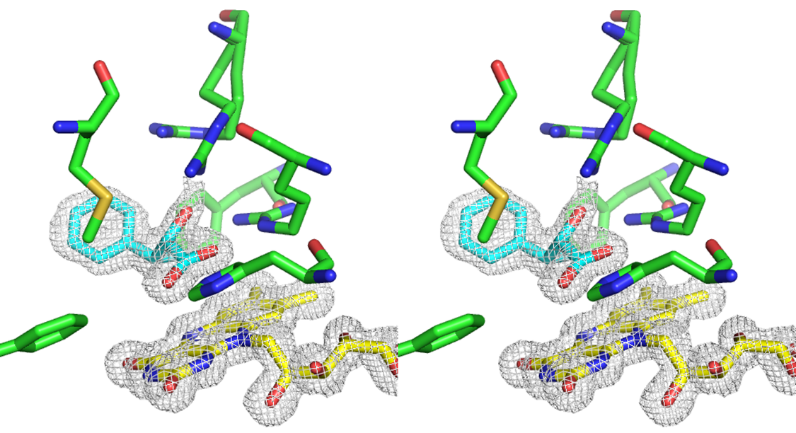


**PDB ID: 6A1N**

**Figure S4. The stereoview of ternary complexes of Hmo/Y128F with fluorinated substrates.** The crystal structure of Hmo in complex with 3,3-difluoro-2-hydroxy-phenylpropanoate (A). The crystal structure of Y128F in complex with 3,3,3-trifluoro-2-hydroxypropanoate (B). The crystal structure of Y128F in complex with 3,3,3-trifluoro-2-dihydroxypropanoate (C). The crystal structure of Y128F in complex with 3,3-difluoro-2-hydroxy-phenylpropanoate (D). The crystal structure of Y128F in complex with 2-fluoro-2-hydroxy-phenylpropanoate (E). Both of the 2F_o_-F_c_ electron density maps are contoured at 1 σ in grey.

**A.**

**
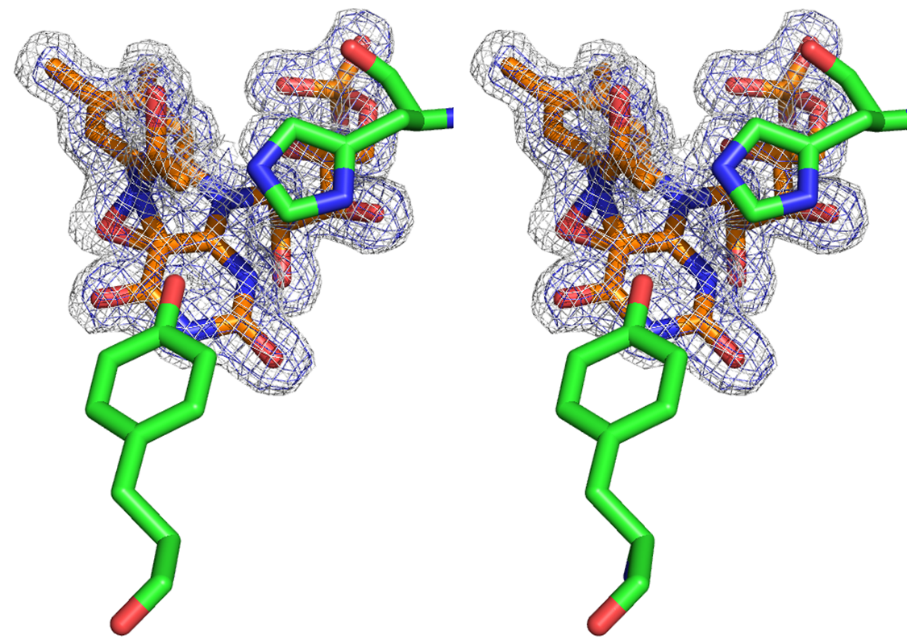
**

**PDB ID: 6A1W**

**B.**


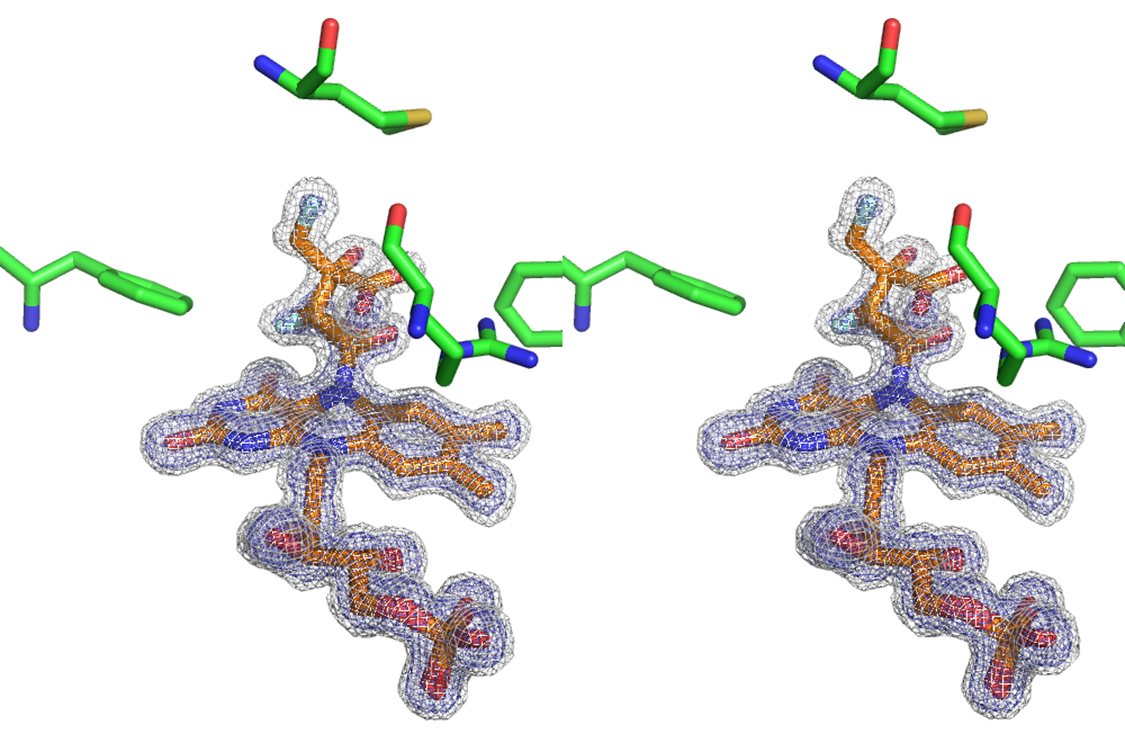


**PDB ID: 6A36**

**C.**

**
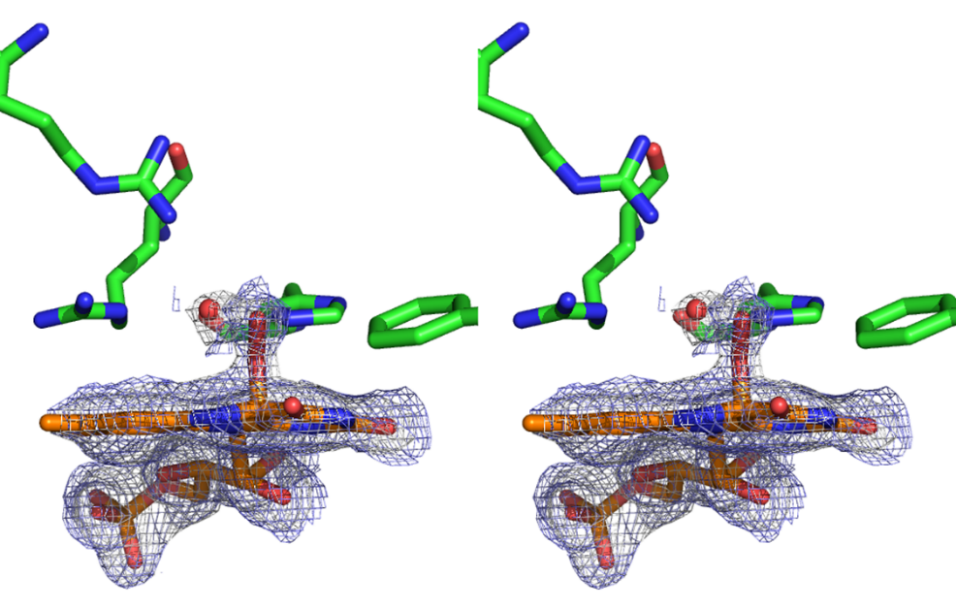
**

**PDB ID: 6A4H**

**D.**


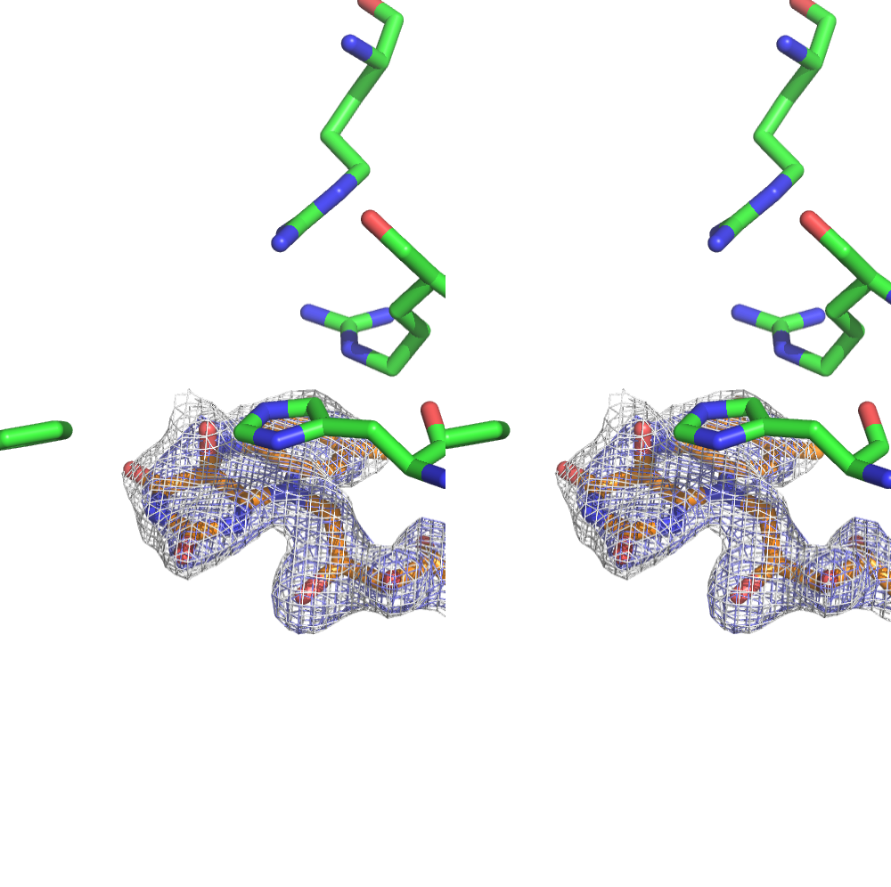


**PDB ID: 6A4G**

**E.**


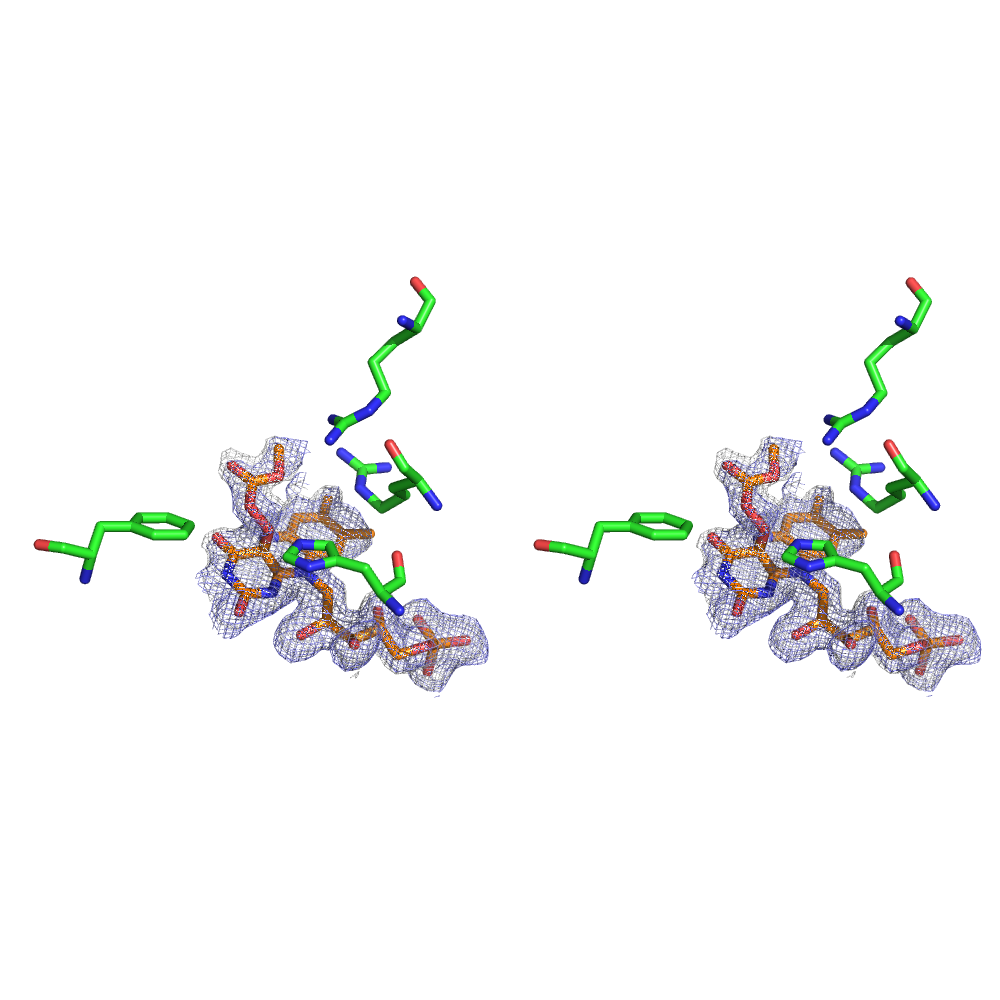


**PDB ID: 6A3D**

**F.**

**
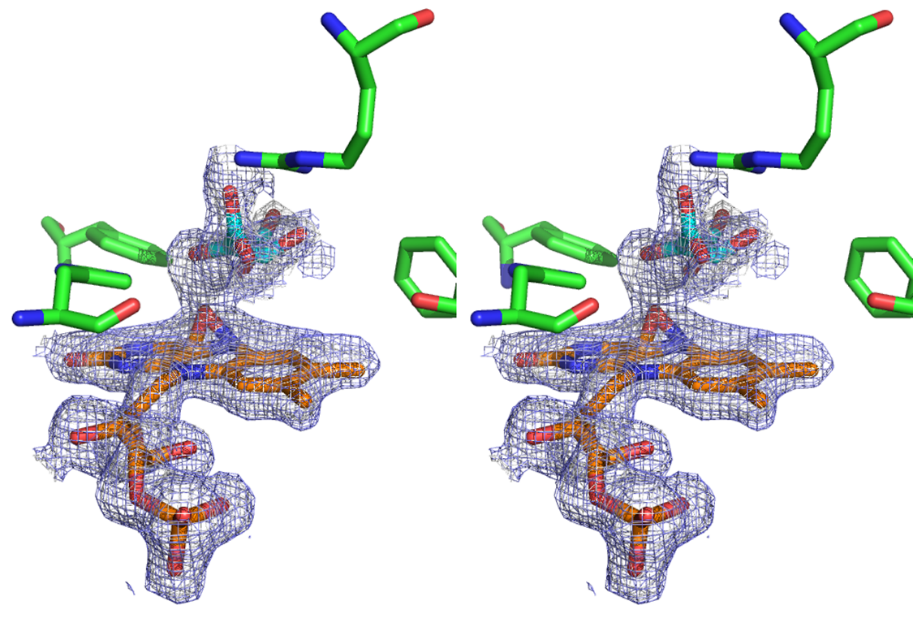
**

**PDB ID: 7BRS**

**Figure S5. The stereoview of C4α-adducts in Hmo/Y128F crystal complexes.** (A) The C4α-N5-epoxide-N5-acetyl-FMN adduct in Y128F. (B) The N5-γ-fluoro-β-hydroxyl,β-carboxyl-butyryl-FMN adduct in Y128F, (C) The C4α-peroxide-FMN adduct in Y128F. (D) The C4α-monooxide-FMN adduct in Y128F (upper panel); superposition of FMN_ox_ (colored magenta), C4α-monooxide-FMN (colored green) and C4α-peroxide-FMN (colored cyan), where C4α of the latter two is of sp^3^ configuration but slightly subject to constrain of neighboring planar atoms. (E) The C4α-methyl ester-peroxide-FMN adduct in Y128F. (F) The C4α-N5-epoxide-FMN adduct in coordination with 2-hydroxyl-3-oxosuccinate in Y128F. The 2F_o_-F_c_ electron density maps are contoured at 1 σ in grey; the simulated annealing omit maps are contoured at 2.5 σ in blue.

C


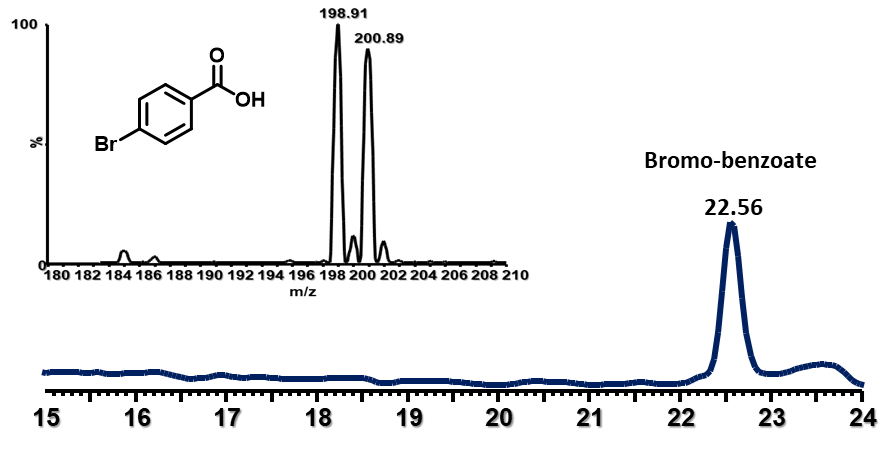


**Figure S6. LC and MS spectra for the enzymatic reaction catalyzed by Y128F against methyl *p*-bromo-(*S*)-mandelate in a H_2_^18^O (80%) buffer solution.** (A) The LC traces of enzymatic reactions catalyzed by Y128F, in which (i) is the LC trace of the substrate methyl *p*-bromo-(*S*)-mandelate (Me *p*-Br-(*S*)-MA) and (ii) is the LC trace of the reaction product *p*-bromo-benzaldehyde (*p*-Br-BA). (B) Electrospray ionization (ESI, negative mode) mass spectrum of *p*-Br-BA, where the intensity ratio for M:M+2:M+4 is about 1:2:1 due to bromine’s two naturally stable isotopes, ^79^Br and ^81^Br. The M+4 indicates that 50% of *p*-Br-BA are incorporated with an ^18^O atom (colored green) from water. (C) The LC trace and mass spectrum of enzymatic reactions catalyzed by Y128F in water solution, where the intensity ratio for M:M+2 is about 1:1.


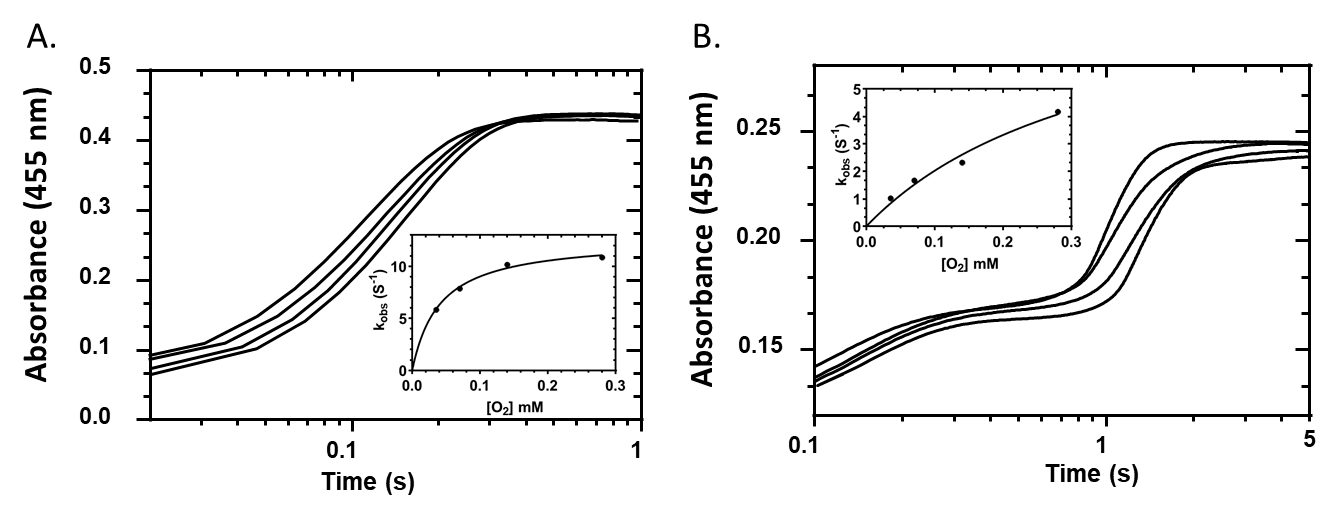


**Figure S7. The rate constants of elimination of H_2_O_2_ or H_2_O for the reduced Hmo/Y128F in reaction with dioxygen.** The kinetics for the elimination of C4α-hydroperoxyflavin was monitored by the absorbance change at 455 nm. Reduced Hmo/Y128F (0.2 mM) was mixed with buffers containing various concentrations of O_2_ (0.035, 0.07, 0.14 and 0.28 mM), and the observed rate constants were calculated on the regression curve of *k*_obs_ of 5.8 s^-1^, 7.9 s^-1^, 10.2 s^-1^, and 10.9 s^-1^ in Hmo (A), and 1.0 s^-1^, 1.7 s^-1^, 2.3 s^-1^ and 3.9 s^-1^ in Y128F (B) against O_2_ concentrations.

**Figure S8. Superimposition of the N5- phenylacetyl adduct over the C4α-peroxide adduct.** The peroxide moiety is sterically compatible with the N5-phenylacetyl moiety, where upon decarboxylation the resulting Cα’-carbanion is within a bonding distance towards the distant oxygen atom of C4α-OO^-^. The complexes of N5-phenylacetyl and C4α-peroxide adducts are colored green and cyan, respectively.


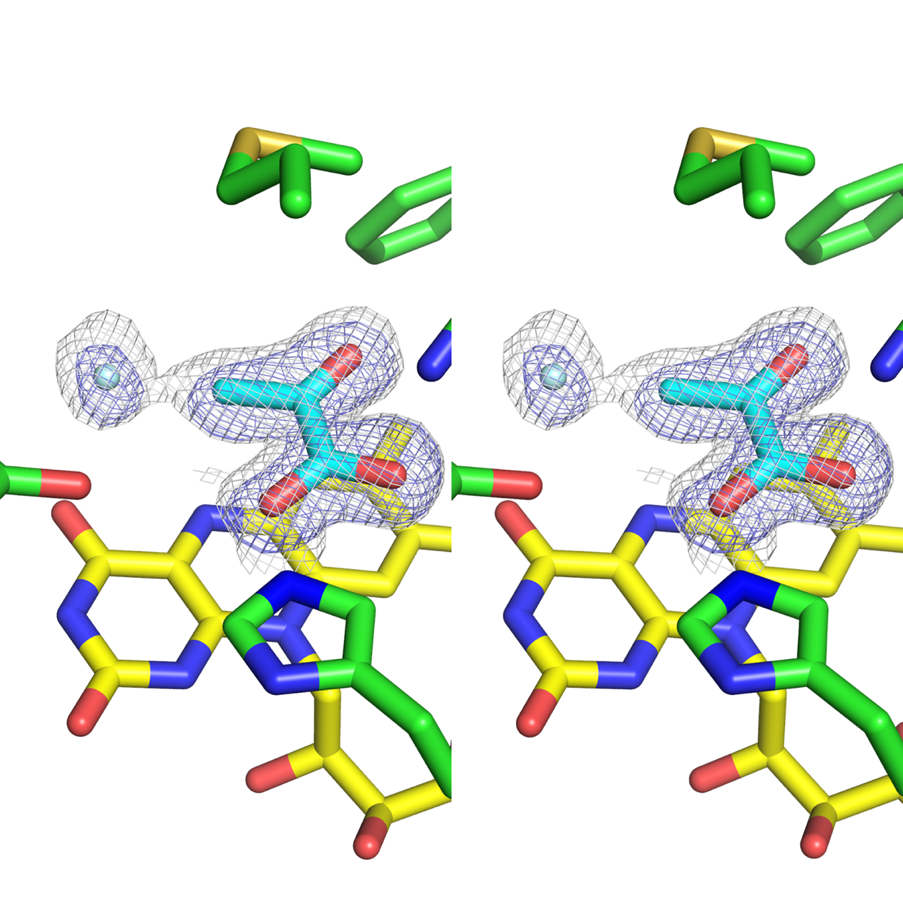


**PDB ID: 6A24**

**Figure S9. The stereoview of monofluoropyruvate in the Hmo ternary complex.** The 2F_o_-F_c_ electron density map and simulated annealing omit map are contoured at 1 and 6 σ, respectively. The active-site residues, cofactor FMN and substrate monofluoropyruvate are colored green, yellow and cyan, respectively, in stick representation.


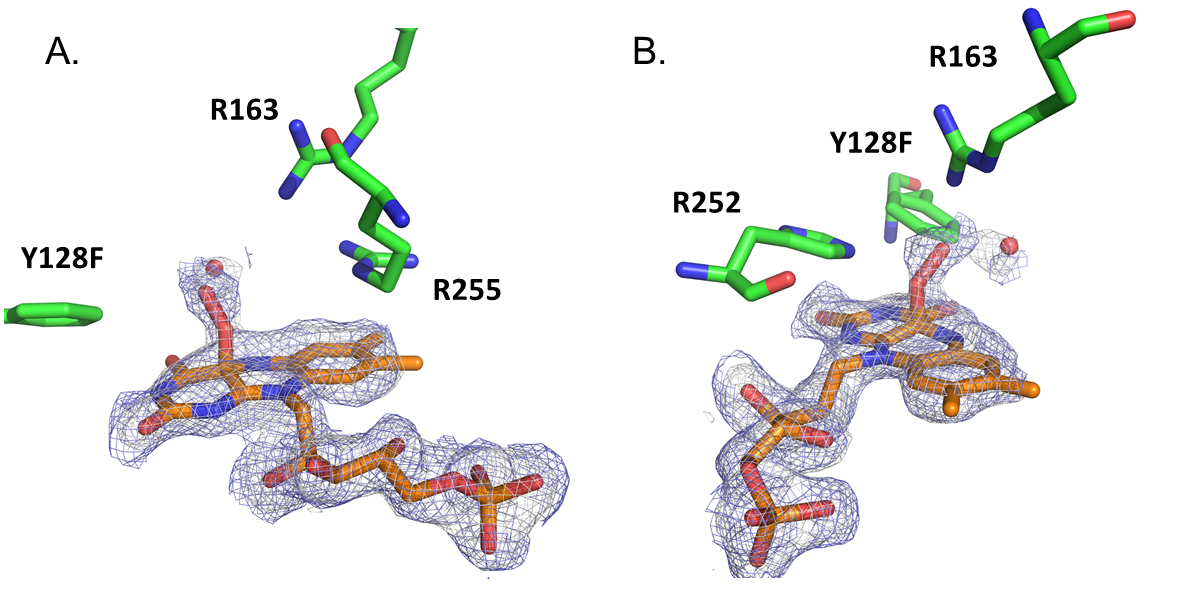


**Figure S10. Views of the C4α-peroxide-FMN adduct from two different orientations.** The 2F_o_-F_c_ electron density map and simulated annealing omit map are contoured at 1 and 2 σ, respectively. The active-site residues and C4α-FMN adduct are colored green and orange, respectively, in stick representation.

C

B

A

**Figure S11. LC traces of β-difluorophenyl lactate catalyzed by Hmo.**A) β-difluorophenyllactate, B) β-difluorophenyllactate can be oxidized by Hmo (Orf22) to β-difluorophenylpyruvate, C) β-difluorophenyllactate is weakly oxidized by Y128F to β-difluorophenylpyruvate.


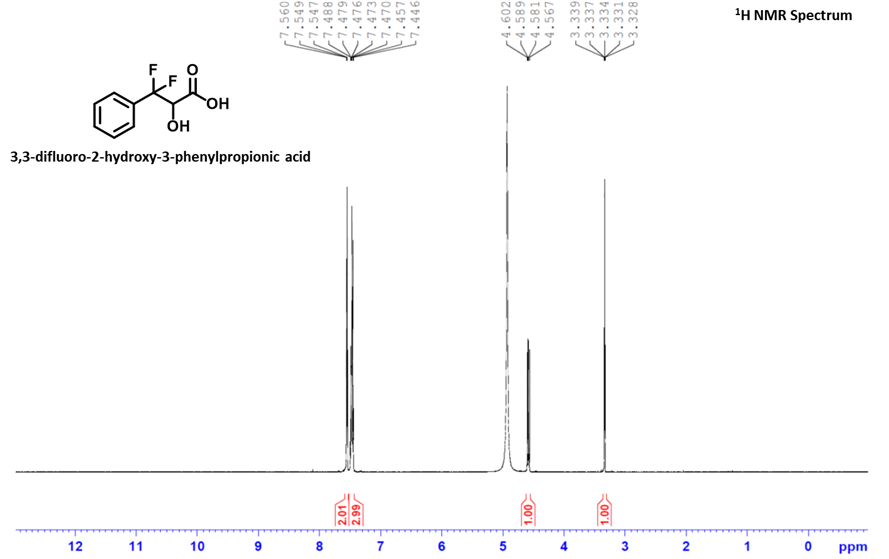


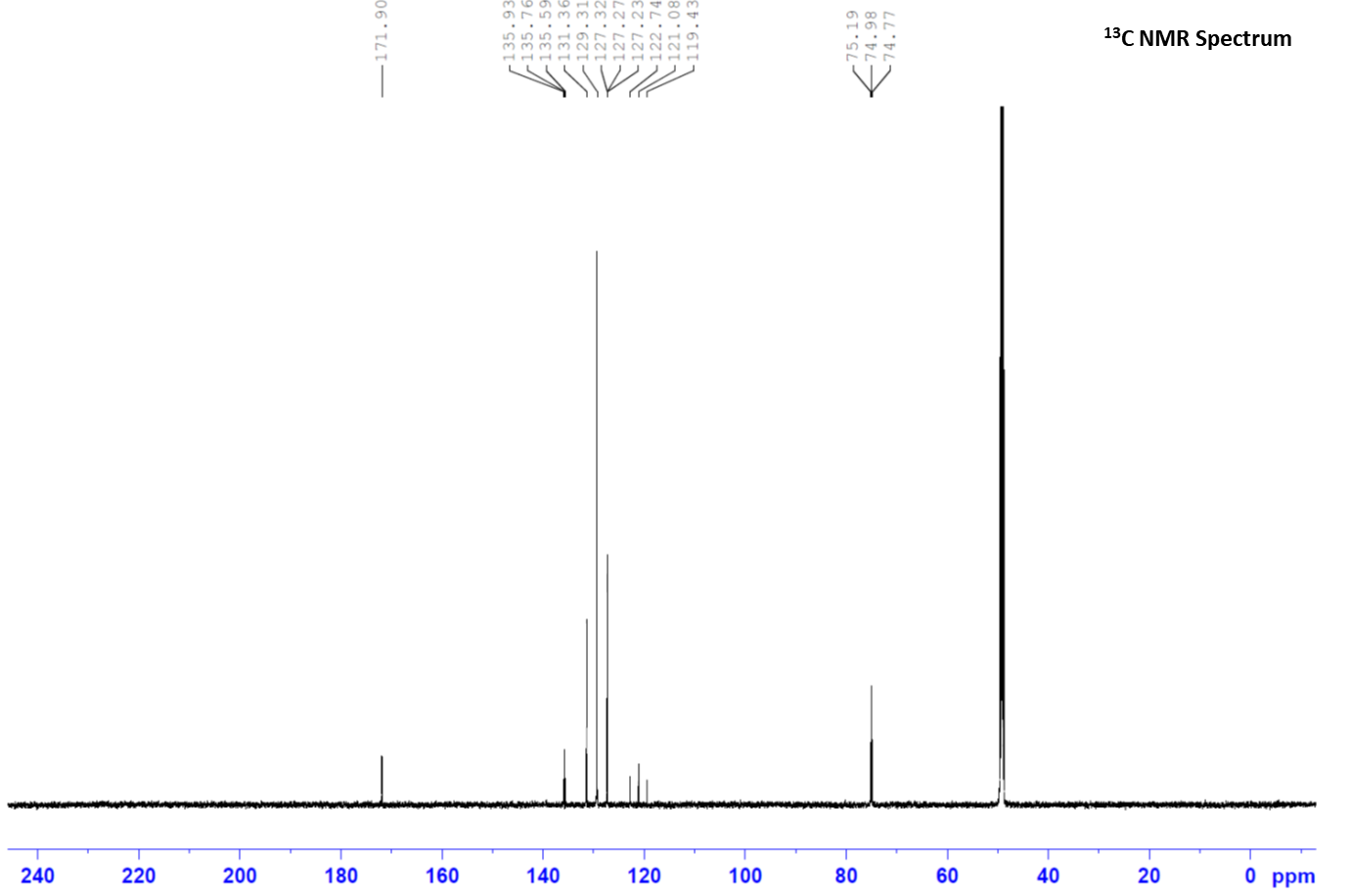


**Figure S12. NMR analyses for 3,3-difluoro-2-hydroxy-3-phenylpropionic acid (β-difluorophenyl lactate).** NMR spectra include ^1^H (top panel), and ^13^C (bottom panel).


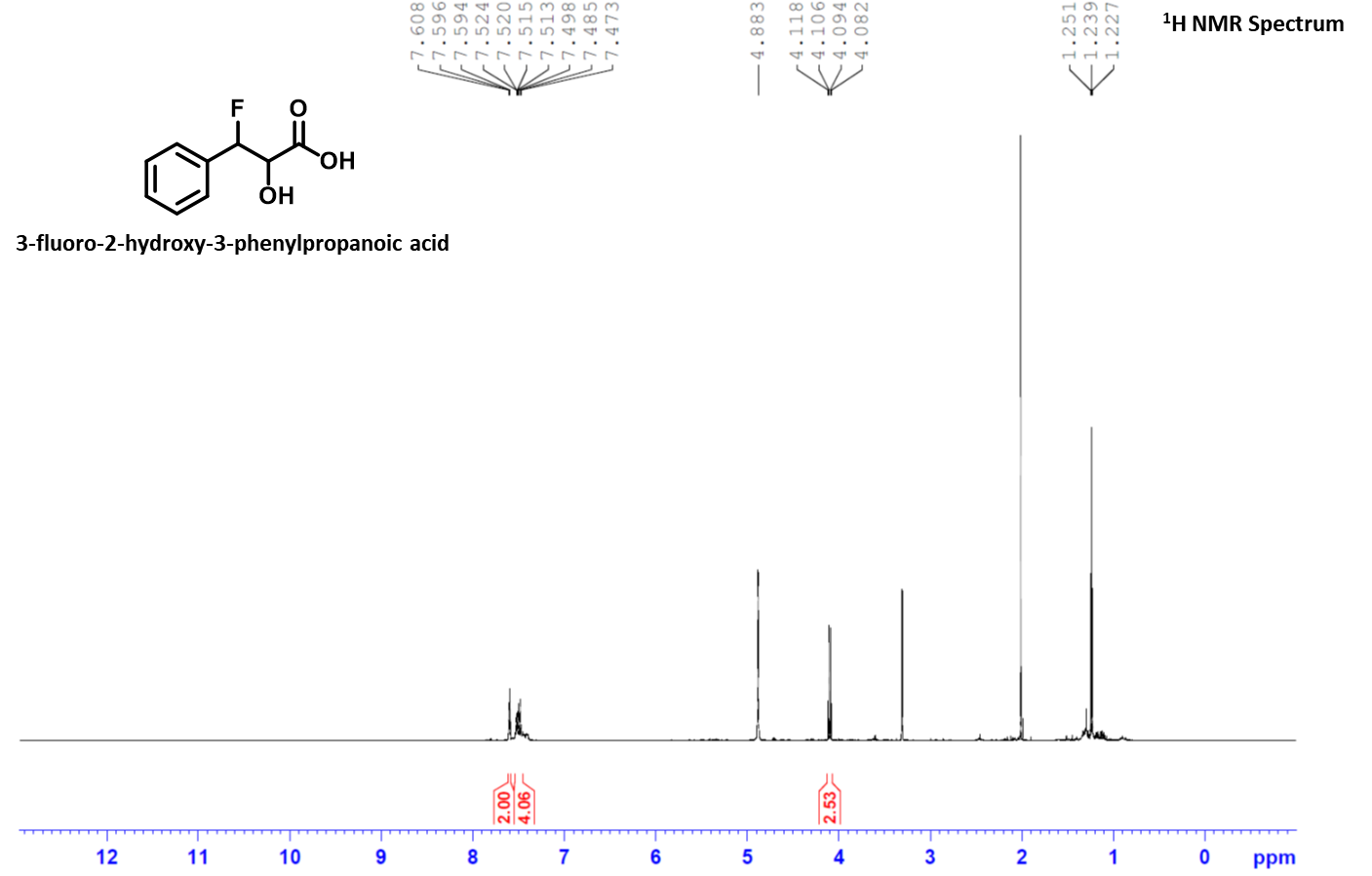


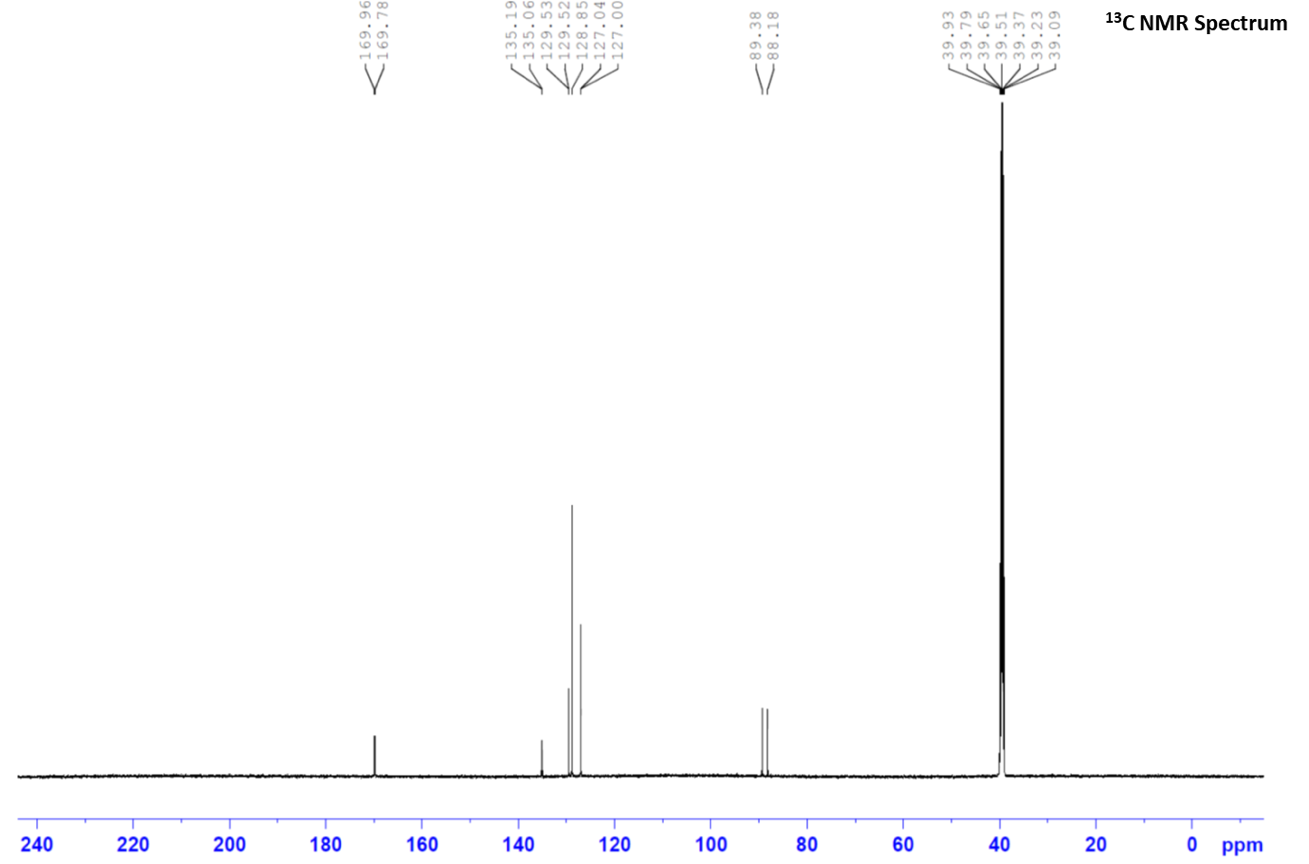


**Figure S13. NMR analyses for 3-fluoro-2-hydroxy-3-phenylpropionic acid (β-fluorophenyl lactate).** NMR spectra include ^1^H (top panel), and ^13^C (bottom panel).

Table S1. Data collection and refinement statistics for Hmo and Y128F

|  | **Hmo_DFHPY** | **Hmo_3FPY** | **Hmo_Enoyl_FMN** | **Y128F_DFHPY** | **Y128F_2FHPY** |
| --- | --- | --- | --- | --- | --- |
| **PDB code** | 5ZZT | 6A24 | 6A1W | 6A01 | 6A1N |
| **Wave length (Å)** | 1.0 | 1.0 | 1.0 | 1.0 | 1.0 |
| **Space group** | *I422* | *I422* | *I422* | *I422* | *I422* |
| ***a*, *b*, *c* (Å)** | 137.7, 137.7, 111.8 | 138.1, 138.1, 111.2 | 137.5, 137.5, 112.2 | 138.2, 138.2, 111.8 | 138.2, 138.2, 111.8 |
| **α, β, γ (°)** | 90, 90, 90 | 90, 90, 90 | 90, 90, 90 | 90, 90, 90 | 90, 90, 90 |
| **Resolution range (Å)^a^** | 30-1.35 (1.40-1.35) | 30-1.39(1.44-1.39) | 30-1.70(1.76-1.70) | 30-1.85(1.92-1.85) | 30-1.42(1.47-1.42) |
| ***R*_merge_ (%)^a, b^** | 4.9 (78.0) | 3.1 (81.0) | 3.7 (65.2) | 4.8 (78.0) | 4.6 (83.0) |
| **〈 *I*/σ(*I*)〉^a^** | 37.7(2.5) | 41.2(2.4) | 34.4(2.4) | 27.4(2.2) | 35.4(2.5) |
| **Completeness (%)^a^** | 99.8(100.0) | 100.0(100.0) | 99.0(99.9) | 99.9(100.0) | 99.9(100.0) |
| **Redundancy^a^** | 12.3(11.1) | 9.6(9.5) | 9.8(9.7) | 10.7(10.2) | 11.8(11.3) |
| **Refinement** |  |  |  |  |  |
| **Resolution range (Å)^a^** | 30-1.35 (1.40-1.35) | 30-1.39(1.44-1.39) | 30-1.70(1.76-1.70) | 30-1.87(1.92-1.87) | 30-1.42(1.47-1.42) |
| **R_work_ (%)^a,^** **^c^** | 17.4 (23.0) | 17.9 (24.5) | 18.1 (24.8) | 16.8 (22.5) | 16.9 (21.8) |
| **R_free_ (%)^a, d^** | 19.0 (25.0) | 19.3 (29.1) | 19.8 (30.6) | 20.0 (26.5) | 18.7 (23.0) |
| **R.m.s. deviations** |  |  |  |  |  |
| **Bond lengths (Å)** | 0.010 | 0.009 | 0.007 | 0.005 | 0.015 |
| **Bond angles (°)** | 1.29 | 1.233 | 1.233 | 0.917 | 1.421 |
| **No. reflections** | 109621 | 102876 | 56862 | 43647 | 97561 |
| **No. atoms** |  |  |  |  |  |
| **Protein** | 2655 | 2711 | 2551 | 2592 | 2603 |
| **Ligand/ion** | 45 | 41 | 36 | 46 | 63 |
| **Water** | 334 | 347 | 313 | 287 | 404 |
| **B-factors** |  |  |  |  |  |
| **Protein** | 20.3 | 19.3 | 20.9 | 25.5 | 19.4 |
| **Ligand/ion** | 15.5 | 20.7 | 18.7 | 20.2 | 31.3 |
| **Water** | 32.1 | 31.5 | 32.9 | 35.8 | 41.0 |

|  | **Y128F_TFHLAC** | **Y128F_TFLAC** | **Y128F_3FPY**  **_FMN** | **Y128F_PEROXI**  **_FMN** | **Y128F_MOXI**  **_FMN** |
| --- | --- | --- | --- | --- | --- |
| **PDB code** | 6A1B | 6A0B | 6A36 | 6A4H | 6A4G |
| **Wave length (Å)** | 1.0 | 1.0 | 1.0 | 1.0 | 1.0 |
| **Space group** | *I422* | *I422* | *I422* | *I422* | *I422* |
| ***a*, *b*, *c* (Å)** | 137.5, 137.5, 112.0 | 137.5, 137.5, 112.5 | 137.7, 137.7, 111.9 | 138.8, 138.8, 107.9 | 139.0, 139.0, 107.2 |
| **α, β, γ (°)** | 90, 90, 90 | 90, 90, 90 | 90, 90, 90 | 90, 90, 90 | 90, 90, 90 |
| **Resolution range (Å)^a^** | 30-1.47(1.52-1.47) | 30-1.65(1.71-1.65) | 30.0-1.44(1.49-1.44) | 30-2.07(2.14-2.07) | 30-1.99(2.06-1.99) |
| ***R*_merge_ (%)^a, b^** | 2.8 (71.2) | 3.7 (58.1) | 2.8 (67.0) | 3.5(59.3) | 4.2(78.4) |
| **〈 *I*/σ(*I*)〉^a^** | 49.2(2.5) | 34.9(3.3) | 52.4(3.0) | 40.6(3.3) | 32.4(2.1) |
| **Completeness (%)^a^** | 99.9(99.9) | 99.6(100.0) | 100.0(100.0) | 99.4(97.7) | 95.3(91.4) |
| **Redundancy^a^** | 11.9(11.1) | 9.6(9.3) | 12.0(11.8) | 9.7(9.6) | 10.1(10.0) |
| **Refinement** |  |  |  |  |  |
| **Resolution range (Å)^a^** | 30-1.47(1.52-1.47) | 30-1.65(1.71-1.65) | 30.0-1.44(1.49-1.44) | 30-1.99(2.06-1.99) | 30-1.99(2.06-1.99) |
| **R_work_ (%)^a,^** **^c^** | 16.3 (23.9) | 15.6 (22.0) | 17.7 (24.7) | 22.7 (27.7) | 22.4 (28.6) |
| **R_free_ (%)^a, d^** | 18.4 (25.3) | 17.3 (21.7) | 19.3 (28.2) | 24.7 (32.5) | 24.5 (33.2) |
| **R.m.s. deviations** |  |  |  |  |  |
| **Bond lengths (Å)** | 0.018 | 0.021 | 0.013 | 0.003 | 0.003 |
| **Bond angles (°)** | 1.508 | 1.983 | 1.670 | 0.695 | 0.703 |
| **No. reflections** | 88157 | 60690 | 89143 | 34562 | 30590 |
| **No. atoms** |  |  |  |  |  |
| **Protein** | 2630 | 2551 | 2542 | 2252 | 2324 |
| **Ligand/ion** | 41 | 40 | 76 | 34 | 33 |
| **Water** | 360 | 389 | 350 | 123 | 126 |
| **B-factors** |  |  |  |  |  |
| **Protein** | 18.4 | 17.4 | 17.3 | 30.8 | 35.0 |
| **Ligand/ion** | 14.6 | 16.2 | 13.1 | 41.2 | 45.8 |
| **Water** | 32.2 | 34.1 | 32.4 | 32.2 | 35.5 |

|  | **Y128F_PEROXI_**  **ME_FMN** | **Y128F_2H3OSA** |  |  |  |
| --- | --- | --- | --- | --- | --- |
| **PDB code** | 6A3D | 7BSR |  |  |  |
| **Wave length (Å)** | 1.0 | 1.0 |  |  |  |
| **Space group** | *I422* | *I422* |  |  |  |
| ***a*, *b*, *c* (Å)** | 138.5, 138.5, 108.5 | 138.4, 138.4, 109.1 |  |  |  |
| **α, β, γ (°)** | 90, 90, 90 | 90, 90, 90 |  |  |  |
| **Resolution range (Å)^a^** | 30-1.92(1.99-1.92) | 30-1.90(1.97-1.90) |  |  |  |
| ***R*_merge_ (%)^a, b^** | 5.0(76.1) | 3.9(74.5) |  |  |  |
| **〈 *I*/σ(*I*)〉^a^** | 33.8(2.0) | 36.4(2.6) |  |  |  |
| **Completeness (%)^a^** | 99.2(92.9) | 98.9 (99.8) |  |  |  |
| **Redundancy^a^** | 9.3(8.0) | 9.7(9.7) |  |  |  |
| **Refinement** |  |  |  |  |  |
| **Resolution range (Å)^a^** | 30-1.92(1.99-1.92) | 30-1.89(1.93-1.89) |  |  |  |
| **R_work_ (%)^a,^** **^c^** | 21.4 (26.8) | 21.5 (30.0) |  |  |  |
| **R_free_ (%)^a, d^** | 24.6 (32.7) | 25.3 (32.3) |  |  |  |
| **R.m.s. deviations** |  |  |  |  |  |
| **Bond lengths (Å)** | 0.007 | 0.015 |  |  |  |
| **Bond angles (°)** | 1.012 | 1.525 |  |  |  |
| **No. reflections** | 38364 | 40611 |  |  |  |
| **No. atoms** |  |  |  |  |  |
| **Protein** | 2436 | 2464 |  |  |  |
| **Ligand/ion** | 38 | 44 |  |  |  |
| **Water** | 165 | 112 |  |  |  |
| **B-factors** |  |  |  |  |  |
| **Protein** | 29.22 | 28.8 |  |  |  |
| **Ligand/ion** | 34.04 | 32.0 |  |  |  |
| **Water** | 31.45 | 24.7 |  |  |  |

^a^Values in parentheses are for the highest resolution shell.

^b^*R*_merge_ ＝Σ|*I_i_*-<*I_i_*>|Σ*I_i_*, *I_i_* is the average intensity value of the equivalent reflections.

^c^*R*_work_ = Σhkl||F_obs_|-|F_calc_||/Σhkl|F_obs_|.

^d^*R*_free_ was calculated from 5% of data randomly excluded data from refinement.

**DFHPY**: 3,3-difluoro-2-hydroxy-phenylpropanoate, **3FPY**: 3-fluoroppyruvate, **FMN**: riboflavin mononucleotide, **Enoyl_FMN**: enoyl-FMN, **2FHPY**: 2-fluoro-2-hydroxy-phenylpropanoate, **TFHLAC**: 3,3,3-trifluoro-2-dihydroxypropanoate, **TFLAC**: 3,3,3-trifluoro-2-hydroxypropanoate, **PEROXI_FMN**: peroxide-FMN, **MOXI_FMN**: monoxide-FMN, **PEROXI_ME_FMN**: peroxide-methyl ester-FMN, **2H3OSA:** 2-Hydroxy-3-oxosuccinate.

**References**

[1] H. W. Yeh, K. H. Lin, S. Y. Lyu, Y. S. Li, C. M. Huang, Y. L. Wang, H. W. Shih, N. S. Hsu, C. J. Wu, T. L. Li, *Acta Crystallogr D Struct Biol* **2019**, *75*, 733-742.
